# RNA-seq reveals differentially expressed genes of rice (*Oryza sativa*) spikelet in response to temperature interacting with nitrogen at meiosis stage

**DOI:** 10.1186/s12864-015-2141-9

**Published:** 2015-11-17

**Authors:** Jun Yang, Xiaorong Chen, Changlan Zhu, Xiaosong Peng, Xiaopeng He, Junru Fu, Linjuan Ouyang, Jianmin Bian, Lifang Hu, Xiaotang Sun, Jie Xu, Haohua He

**Affiliations:** Key Laboratory of Crop Physiology, Ecology and Genetic Breeding, Ministry of Education, College of Agronomy, Jiangxi Agricultural University, 1101 Zhimin Street, Changbei economic and technological development zone, QingShanHu District, Nanchang, Jiangxi Province 330045 China

**Keywords:** *De novo* assembly, Differentially expressed genes, High temperature, Nitrogen, Rice, RNA-seq, Spikelet fertility, Transcriptome

## Abstract

**Background:**

Rice (*Oryza sativa*) is one of the most important cereal crops, providing food for more than half of the world’s population. However, grain yields are challenged by various abiotic stresses such as drought, fertilizer, heat, and their interaction. Rice at reproductive stage is much more sensitive to environmental temperatures, and little is known about molecular mechanisms of rice spikelet in response to high temperature interacting with nitrogen (N).

**Results:**

Here we reported the transcriptional profiling analysis of rice spikelet at meiosis stage using RNA sequencing (RNA-seq) as an attempt to gain insights into molecular events associated with temperature and nitrogen. This study received four treatments: 1) NN: normal nitrogen level (165 kg ha^−1^) with natural temperature (30 °C); 2) HH: high nitrogen level (330 kg ha^−1^) with high temperature (37 °C); 3) NH: normal nitrogen level and high temperature; and 4) HN: high nitrogen level and natural temperature, respectively. The *de novo* assembly generated 52,553,536 clean reads aligned with 72,667 unigenes. About 10 M reads were identified from each treatment. In these differentially expressed genes (DEGs), we found 151 and 323 temperature-responsive DEGs in NN-vs-NH and HN-vs-HH, and 114 DEGs were co-expressed. Meanwhile, 203 and 144 nitrogen-responsive DEGs were focused in NN-vs-HN and NH-vs-HH, and 111 DEGs were co-expressed. The temperature-responsive genes were principally associated with calcium-dependent protein, cytochrome, flavonoid, heat shock protein, peroxidase, ubiquitin, and transcription factor while the nitrogen-responsive genes were mainly involved in glutamine synthetase, transcription factor, anthocyanin, amino acid transporter, leucine zipper protein, and hormone. It is noted that, rice spikelet fertility was significantly decreased under high temperature, but it was more reduced under higher nitrogen. Accordingly, numerous spikelet genes involved in pollen development, pollen tube growth, pollen germination, especially sporopollenin biosynthetic process, and pollen exine formation were mainly down-regulated under high temperature. Moreover, the expression levels of co-expressed DEGs including 5 sporopollenin biosynthetic process and 7 pollen exine formation genes of NN-vs-NH were lower than that of HN-vs-HH. Therefore, these spikelet genes may play important roles in response to high temperature with high nitrogen and may be good candidates for crop improvement.

**Conclusions:**

This RNA-seq study will help elucidate the molecular mechanisms of rice spikelet defense response to high temperature interacting with high nitrogen level.

**Electronic supplementary material:**

The online version of this article (doi:10.1186/s12864-015-2141-9) contains supplementary material, which is available to authorized users.

## Background

Rice (*Oryza sativa* L.) is the staple food for more than half of the world’s population, and a continuous increase in rice production is needed to meet the growing food demand resulted from population growth in the future [1]. In the recent hundred of years, rice is increasingly cultivated in more marginal environmental stresses because of global warming, and heat stress is a major abiotic limitation to plant growth and development [2]. The Intergovernmental Panel on Climate Change (IPCC) predicted that the vulnerability of the crop will be increased with a projected global average surface temperature increase by 0.74 ± 0.18 °C [3]. In these environments, the uncontrollable temperature frequently exceed the critical temperature of seed set, resulting in spikelet sterility and reduced yield [4, 5]. It was estimated that rice grain yields decline by 10 % for each 1 °C increase in minimum temperature [6]. In addition to the temperature, the controllable nitrogen (N) is another factor affecting rice yield. In the past, increased rice production was largely attributed to the elevated application of nitrogen [7]. Moreover, grain yields were challenged by high temperature interacting with other abiotic stresses [8, 9]. But few reports are demonstrated the effects of high temperature interacting with nitrogen on rice production at meiosis stage.

High temperature beyond the critical threshold at reproductive stage leads to spikelet sterility. Morphological observations revealed that reduced spikelet fertility was mainly due to poor anther dehiscence, low pollen production and pollen viability, and reduced germination rate on the rice stigma under high temperature [4, 5, 10, 11]. The recent studies about the molecular biology of spikelet fertility had shown that many differentially expressed genes (DEGs) played an essential role in heat response. It was reported that high temperature treatment (39 °C) at the microspore stage reduced spikelet fertility, DNA microarray analysis revealed that at least 13 genes were designated as high temperature-repressed genes in the anther, and these genes expressed specifically in the immature anther were mainly in the tapetum at the microspore stage and down-regulated after 1 d of high temperature [11]. Zhang et al. [12] provided a gene expression profile of rice panicle grown under 40 °C for different durations (0 min, 20 min, 60 min, 2 h, 4 h, and 8 h) during anther development by using a rice microarray, and found that the identified differentially expressed genes were mainly involved in transcriptional regulation, transport, cellular homeostasis, and stress response. Furthermore, the time-dependent gene expression pattern was discovered under heat stress, and the regulation model central to reactive oxygen species (ROS) combined with transcriptome and ROS quantification data in rice panicle indicated the great importance to maintain ROS balance and the existence of wide cross-talk in heat response in their study. It has been reported that 50.4 % of all genes in the rice genome (28,296/56,143) were expressed at 25 °C, and a similar number of genes (50.2 %; 28,189/56,143) were expressed in rice grown under 30 °C. Furthermore, the temperature markedly stimulated several super-families of transcription factors, including bZIP, MYB, and WRKY [13]. Thus far, little is known about the transcriptional mechanism of rice spikelet in response to heat at meiosis stage.

Nitrogen is an essential components of various macromolecules, such as proteins, nucleic acids, many cofactors, and some plant hormones [14]. Nitrogen affects many aspects of plant function, from metabolism to resource allocation, growth, and development [15, 16]. In order to meet the high nitrogen requirement of plant growth and crop production, a large quantity of nitrogen fertilizers are applied [14, 17]. However, crop plants only use less than half of the applied nitrogen fertilizers [18], and the unused nitrogen (nitrogen pollution) is inevitably becoming a threat to global environment [19, 20]. Furthermore, the more application of nitrogen fertilizers has markedly increased the cost in crop production, which greatly affects the income of the farmers. Thus, it is extremely valuable to develop strategies that crops are less dependent on the heavy application of nitrogen fertilizers to create a sustainable and efficient agricultural system while maintaining high yield. Efforts have been directed to understanding the molecular basis of plant responses to nitrogen, and many nitrogen-responsive genes were identified [21]. Ammonium (NH_4_^+^) and nitrate (NO_3_^−^) are two primary inorganic nitrogen sources available for plants. Nitrate and ammonium are absorbed by the plant via a variety of transporters that are divided into high-affinity transport systems (HATS) and low-affinity transport systems (LATS) [15, 16]. Nitrate is taken up by the low and high affinity nitrate transporter gene family members (NRT1 and NRT2), reduced to nitrite by nitrate reductase (NR), and to ammonium by nitrite reductase (NiR) [22]. It is well known that the members of AMT1 gene family encoded the HATS for ammonium [23]. Ammonium is incorporated into organic molecules, catalyzed primarily by glutamine synthetase (GS) and glutamate synthase (GOGAT) pathway [24]. At the molecular level, nitrogen limitation altered the expression levels of 629 genes with 340 of them up-regulated and 289 of them down-regulated in *Arabidopsis*, and numerous nitrogen-responsive genes encoded transcription factors, signal transduction components, and proteins required for hormone synthesis and response [25]. It was reported that 10,422 genes at an early stage of low nitrogen stress in rice seedling were identified [26]. Wang et al. [27] studied the response of seedlings grown on ammonium to the addition of low or high levels of nitrate, and identified 25 and 49 nitrogen-responsive genes to low or high nitrate induction, respectively. These studies have provided valuable insights into nitrogen response and its linkage to other biological pathways. However, knowledge about plant genes and pathways in response to nitrogen is still lacking, while such knowledge is essential for formulating strategies for manipulating the genetic architecture of the plants to improve the nitrogen use efficiency.

The combination of nitrogen and temperature affects plant growth and development. Weng at al [28] examined the differences between photosynthetic activity and dark respiratory rate as influenced by leaf nitrogen levels and temperatures in rice, and found the photosynthetic rates and respiratory rate were correlated with the leaf nitrogen content. Liu et al. [29] evaluated mycorrhizal rice growth based on treatments at two temperatures (15 °C and 25 °C) and two nitrogen levels (20 mg pot^−1^ and 50 mg pot^−1^). The arbuscular mycorrhizal fungi (AMF) colonization of rice resulted in different responses of the plants to low and high nitrogen levels. Satake et al. [30] showed that high nitrogen level from the spikelet differentiation stage to the young microspore stage greatly increased the sensitivity to coolness at the critical stage in rice plants. Hayashi [31] showed that cool temperature (12 °C) for 3 days decreased rice spikelet fertility by 36 % under standard-nitrogen and 42 % under high-nitrogen conditions. But there are no reports about the effects of the nitrogen interacting with high temperature on spikelet fertility in rice production.

Recently, RNA sequencing technology, based on next-generation sequencing technology, provides a platform for measuring differences in gene expression [32]. Unlike microarrays, RNA-seq does not require prior knowledge of gene sequences. Furthermore, RNA-seq provides a far more precise measurement of transcripts and has been successfully used for transcript profiling in many plant species [33, 34]. Seed setting rate, one of the most important components of rice grain yields, is significantly affected by high temperature during pollen mother cell meiosis. However, limited information about differentially expressed genes of rice spikelet in response to high temperature is available at meiosis stage. Moreover, no reports demonstrate that nitrogen level can regulate the effects of high temperature at meiosis stage on rice production. In this study, we used Illumina RNA-seq technology to profile different gene expression of rice spikelet from a conventional strain Zhong531 treated by high temperature and high nitrogen level at meiosis stage. The overall objective of this study was to increase our understanding of the heat response in rice spikelet and provided good candidate genes for crop improvement.

## Methods

### Plant material

One rice (*Oryza sativa* L. ssp. *indica*) strain, Zhong531, was chosen for this study. The trials were conducted between March and August in 2014, in net house at High-Tech Agricultural Science and Technology Park of Jiangxi Agricultural University (latitude: 28° 46′ N, longitude: 115° 50′ E, altitude: 48.80 m), Nanchang, Jiangxi Province, China. Seed dormancy was broken by exposure to the sun for 3 d, followed by pre-germination and sown in a rice field. Thirty-day-old seedlings were then transplanted on 25 April at a spacing of 22.5 cm × 24.5 cm with only one seedling per hill. The physical and chemical properties of experimental soil were as follows: soil pH, organic matter, total nitrogen, available nitrogen, available phosphorous (P), and available potassium (K) were 5.94, 28.72 g kg^−1^, 1.45 g kg^−1^, 92.01 mg kg^−1^, 28.31 mg kg^−1^, and 221.67 mg kg^−1^, respectively. The experimental received four treatments: 1) NN: normal nitrogen level (165 kg N ha^−1^, as the control) with natural temperature (30 °C, as the control); 2) HH: high nitrogen level (330 kg N ha^−1^) with high temperature (37 °C); 3) NH: normal nitrogen level and high temperature; and 4) HN: high nitrogen level and natural temperature, respectively. The amount of nitrogen fertilizer in the form of urea for the normal nitrogen level was recommended by local agricultural extension employees based on experience and target yield. For normal nitrogen level, 66, 33, and 66 kg N ha^−1^ (as pure N) were applied at basal (2 d before transplanting), tillering (10 d after transplanting), and panicle initiation (30 d after transplanting), respectively. For high nitrogen level, 66, 66, and 198 kg N ha^−1^ were applied at basal, tillering, and panicle initiation, respectively [35]. Phosphorus (90 kg P ha^−1^ as P_2_O_5_) was applied 2 d before transplanting. Potassium (180 kg K ha^−1^ as K_2_O) was applied in two equal splits at basal and panicle initiation. Weeds in the field were manually removed at early growth stages. Insecticides were used to prevent insect damage. All other agronomic practices referred to the local recommendations to avoid yield loss.

### High temperature treatment

Four temperature-controlled growth cabinets were specifically designed to study the impacts of the high temperature (37 °C) and natural temperature (30 °C), respectively. Each cabinet (1.91 m × 0.76 m × 1.83 m in length, width and height, respectively) was fixed at a 5 m interval to ensure adequate ventilation. For high temperature analysis, rice plants along with soil at the female stamen primordium differentiation stage were randomly transplanted into plastic pots to adapt for 3 d and then transferred to growth cabinets. The pot with a hole at the bottom has internal diameter of 17 cm and height of 16 cm, and the rice plants transferred into pots grew normally and stably without withered and yellow leaves. Twelve rice plants with each in a pot for each treatment combination at the formation stage of pollen mother cell [11] were randomly exposed to high temperature (from 8:00 o'clock to 18:00 o'clock, Beijing time, 37 °C; from 18:00 o'clock to the next morning 8:00 o'clock, 30 °C), and natural temperature (from 8:00 o'clock to 18:00 o'clock, 30 °C; from 18:00 o'clock to the next morning 8:00 o'clock, 25 °C) for consecutive 4 d, respectively [5]. Two stand-alone sensors were placed in the rice canopy near the panicle base (few centimeters into the canopy) in each cabinet to measure temperature once 15-min interval, with all the sensors connected to data loggers (HOBO, U22–001, USA) [36, 37]. The other controlled environmental conditions (white fluorescent illumination of 540 μmol m^−2^ s^−1^ day/ 0 μmol m^−2^ s^−1^ night, and relative humidity of 75 % day/ 80 % night) in each growth cabinet were consistent. The four treatments of combining temperature and nitrogen were in two repeats, each repeat with six plants (pots) per growth cabinet. No cabinet or replicate effects were observed, therefore, data collected from different replicates and cabinets for each treatment were pooled. According to the descriptions by Endo et al. [11], Tang et al. [38], and Chen et al. [39], we collected young florets in the same phases of the meiosis stage as samples for further analysis. Features used to distinguish this stage are as follows: a) rice was grown for about 2 months, 13 to19 days before flowering; b) the distance between the auricle of the flag leaf and that of the penultimate leaf was within 1 cm; c) the young panicle length was about 2 cm; d) the floret length was 2 to 3 mm. Young florets during the meiosis of pollen mother cell at the middle of main panicles after 4 d in the high temperature environments were collected, frozen in liquid nitrogen immediately, and stored at −80 °C for further use. Each sample represented two replicates (each replicate had 3 plants).

After high temperature, the pots were removed and the remaining 6 undamaged rice plants were returned to natural field conditions until seed maturity. The average temperatures in the cabinets were close to the required targets: high temperature (from 8:00 o'clock to 18:00 o'clock, actual, 37.08 °C, SE = ±0.13 °C; from 18:00 o'clock to the next morning 8:00 o'clock, 29.71 ± 0.05 °C), and natural temperature (from 8:00 o'clock to 18:00 o'clock, 29.46 ± 0.11 °C; from 18:00 o'clock to the next morning 8:00 o'clock, 26.07 ± 0.13 °C), respectively.

### Spikelet fertility (seed-set)

Spikelet fertility (seed-set) was estimated according to the procedures of Prasad et al. [4]. At physiological maturity stage, 16 randomly selected main tiller panicles (four each from separate plant) were tagged and harvested in each treatment. Spikelet fertility was estimated as the ratio of the number of filled grains to the total number of reproductive sites (florets) and expressed as percentage. Each floret was pressed between the forefinger and thumb to determine if the grain was filled or not. Number of filled grains included both completely and partially filled grains.

### Statistical analysis

Differences among the means of spikelet fertility in the four treatments were analyzed using Student’s *t*-test (SPSS statistical software ver. 17.0). The standard errors of the mean were also calculated and presented in the graphs as error bars.

### cDNA preparation for Illumina sequencing

Total RNA was extracted using TRIzol reagent (Invitrogen) according to the manufacturer’s instructions. The total RNA samples from the four treatments were mixed and pooled (with equal amount of RNA from each treatment) as one sample for transcriptome sequencing (paired ends sequencing, 90 bp) to obtain as much gene expression information as possible, but they were subjected individually to conduct digital gene expression (DGE) sequencing. The total RNA samples were first treated with deoxyribonuclease I (DNase I) to degrade any possible DNA contamination. Then the mRNA was enriched by using the Oligo(dT) magnetic beads. Mixed with the fragmentation buffer, the mRNA was fragmented into short fragments (about 200 bp). Then the first strand of cDNA was synthesized by using random hexamer-primer. Buffer, dNTPs, RNase H and DNA polymerase I were added to synthesize the second strand. The double strand cDNA was purified with magnetic beads. End reparation and 3-end single nucleotide A (adenine) addition was then performed. Finally, sequencing adaptors were ligated to the fragments. The fragments were enriched by PCR amplification. During the quality control (QC) step, Agilent 2100 Bioanaylzer, and ABI StepOnePlus Real-Time PCR System were used to qualify and quantify of the sample library. The library products were ready for sequencing via Illumina HiSeq™ 2000 at the Beijing Genomics Institute (BGI, http://www.genomics.cn/index; Shenzhen, China) (Additional file 1: Figure S1). Image data output from sequencing machine were transformed by base calling into sequence data, called raw data or raw reads, and was stored in fastq format.

### Data filtering and *de novo* assembly

Raw reads produced from sequencing machines contained dirty reads which contain adapters, unknown or low quality bases. These data would negatively affect following bioinformatics analysis. After removal of adaptor sequences along with low quality reads (the rate of reads which quality value < = 10 is more than 20 %), and reads of larger than 5 % unknown sequences (reads with unknown sequences ‘N’), the resting clean reads were assembled into unigenes by Trinity [40], a short-read assembly program.

Transcriptome *de novo* assembly was carried out with a short reads assembling program–Trinity. Trinity combined three independent software modules: Inchworm, Chrysalis, and Butterfly, was applied sequentially to process large volumes of RNA-seq reads. The Trinity software first combined reads with a certain length of overlap to form longer fragments without N, forming contigs. Then the reads were mapped back to contigs, with paired-end reads it was able to detect contigs from the same transcript as well as the distances between these contigs. Then, Trinity connected these contigs to get consensus sequences that contained the least N and could not be extended on either end. Such sequences were defined as unigenes. When multiple samples from the same species were sequenced, unigenes from each sample’s assembly can be taken into further process of sequence splicing and redundancy removing with sequence clustering software to acquire non-redundant unigenes as long as possible. Then gene family clustering was done. The unigenes would be divided to two classes. One was clusters, which the prefix was CL and the cluster ID was behind. In the same cluster, there were several unigenes with the similarity between them was more than 70 %. And the other was singletons, which the prefix was unigene.

In the final step, blastx alignment (evalue < 0.00001) between unigenes and protein databases like NR (NCBI non-redundant protein sequences), Swiss-Prot (A manually annotated and reviewed protein sequence database), KEGG (Kyoto Encyclopedia of Genes and Genomes) and COG (Cluster of Orthologous Groups of proteins) was performed, and the best aligning results were used to decide sequence direction of unigenes. If results of different databases conflicted with each other, a priority order of NR, Swiss-Prot, KEGG and COG should be followed when deciding sequence direction of unigenes. When a unigene happened to be unaligned to any of the above four databases, the software named ESTScan [41] would be introduced to decide its sequence direction. For unigenes with sequence directions, we provided their sequences from 5' end to 3' end; for those without any direction we provided their sequences from the assembly software (Additional file 2: Figure S2).

### Statistics of digital gene expression (DGE) sequencing

After DGE sequencing, clean reads were mapped to reference genes (*de novo* assembly) and/or reference genome (*Indica* rice database) using SOAPaligner/ SOAP2 [42]. No more than 2 mismatches were allowed in the alignment. The distribution of reads on the reference genes was used to evaluate the randomness [34]. Genes with similar expression patterns usually indicated functional correlation. We performed a cluster analysis of gene expression patterns with cluster software [43] and Java Treeview software [44]. Further evaluation included quality assessment of reads, and sequencing saturation analysis, gene sequencing coverage, correlation analysis of all genes between every two samples replicates.

### Functional unigene annotation and COG classification

Unigene annotation provides information of expression and functional annotation of a unigene. Information of functional annotation gives protein functional annotation, COG functional annotation and Gene Ontology (GO) functional annotation of unigenes. Unigene sequences were firstly aligned to protein databases like NR, Swiss-Prot, KEGG and COG (e-value < 0.00001) by BLASTX, and NCBI nucleotide sequences database (NT) (e-value < 0.00001) by BLASTN, retrieving proteins with the highest sequence similarity with the given unigenes along with their protein functional annotations (Additional file 3: Figure S3).

We can get GO functional annotation with NR annotation. GO is an international standardized gene functional classification system which offers a dynamic-updated controlled vocabulary and a strictly defined concept to comprehensively describe properties of genes and their products in any organism. GO has three ontologies: molecular function, cellular component and biological process. The basic unit of GO is GO-term. Every GO-term belongs to a type of ontology. With NR annotation, we used Blast2GO [45] program to get GO annotation of unigenes. Blast2GO has been cited by other articles for more than 150 times and is widely recognized a GO annotation software. After getting GO annotation for every unigene, we used WEGO [46] software to do GO functional classification for all unigenes and to understand the distribution of gene functions of the species from the macro level. GO enrichment analysis provides all GO terms that significantly enriched in DEGs comparing to the genome background, and filter the DEGs that correspond to biological functions. This method firstly mapped all DEGs to GO terms in the database (http://www.geneontology.org/), calculating gene numbers for every term, then using hypergeometric test to find significantly enriched GO terms in DEGs comparing to the genome background. The calculating formula is:

$$ \mathrm{P}\kern0.5em =\kern0.5em 1-\kern0.5em \underset{i\kern0.5em =\kern0.5em 0}{\overset{m\kern0.5em -\kern0.5em 1}{\varSigma }}\frac{\left({}_i^M\right)\left({}_{i\kern0.5em -\kern0.5em i}^{N\kern0.5em -M}\right)}{\left({}_n^N\right)} $$

Where N is the number of all genes with GO annotation; n is the number of DEGs in N; M is the number of all genes that are annotated to the certain GO terms; m is the number of DEGs in M. The calculated P-value goes through Bonferroni Correction, taking corrected P-value < = 0.05 as a threshold. GO terms fulfilling this condition are defined as significantly enriched GO terms in DEGs.

KEGG (Kyoto Encyclopedia of Genes and Genomes) is the major public pathway-related database [47, 48]. Pathway enrichment analysis identifies significantly enriched metabolic pathways or signal transduction pathways in DEGs comparing with the whole genome background. The calculating formula was the same as that in GO analysis.

COG (Cluster of Orthologous Groups of proteins) is a database which classifies orthologous gene product, each COG protein is presumed coming from ancestral protein, the construction of COG database is based on the complete genome encoding protein and phylogenetic relationships of bacteria, algae, eucaryon. We mapped the unigene to the COG database, predicted the possible functions and statistics, understand gene function distribution characteristics of the species from the macro [49].

### Screening of differentially expressed genes (DEGs)

The expression level of unigene was calculated by using RPKM method [50] (Reads Per kb per Million reads), and the formula is shown as follows:

$$ RPKM\kern0.5em =\kern0.5em \frac{10^6C}{NL/{10}^3} $$

Here RPKM (A) is the expression level of gene A, C is number of reads that uniquely aligned to gene A, N is total number of reads that uniquely aligned to all genes, and L is number of bases of gene A. The RPKM method is able to eliminate the influence of different gene length and sequencing discrepancy on the calculation of gene expression level. Therefore, the RPKM values can be directly used for comparing the difference of gene expression among samples.

DEGs analysis included the screening of genes that were differentially expressed among samples, and GO functional enrichment analysis and KEGG pathway enrichment analysis for these DEGs. Referring to the method of Audic and Claverie [51], we had developed a strict algorithm to identify DEGs between two samples. P-value corresponded to differential gene expression test. FDR (False Discovery Rate) is a method to determine the threshold of P-value in multiple tests. Assume that we had picked out R differentially expressed genes in which S genes really showed differential expression and the other V genes were false positive. If we decided that the error ratio Q = V / R must stay below a cutoff (<1 %), we should preset the FDR to a number no larger than 0.01 [52]. We used FDR ≤0.001 and the absolute value of log_2_ (Ratio) ≥1 as the threshold to judge the significance of gene expression difference. More stringent criteria with smaller FDR and bigger fold-change value can be used to identify DEGs (Additional file 4: Figure S4).

## Results

### Illumina paired-end sequencing and *de novo* assembly of transcriptome

**Fig. 1:**
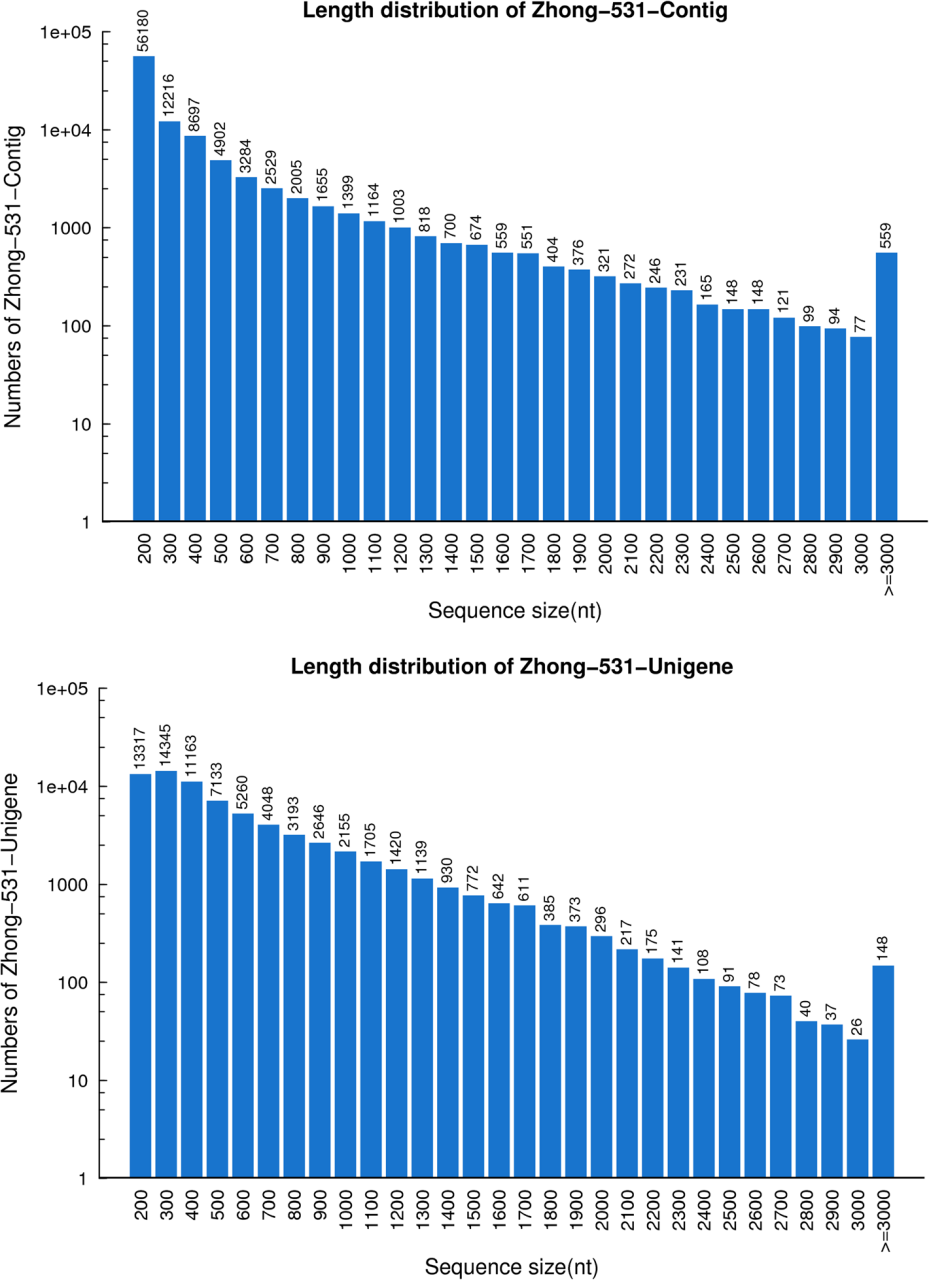
The length distribution of contigs and unigenes. The horizontal coordinates are contig lengths or unigenes lengths, and the vertical coordinates are numbers of contigs or unigenes

Illumina paired-end sequencing generated a total of 61,733,120 raw reads (Table 1). After filtration, 52,553,536 clean reads with accumulated length of 4,729,818,240 bp were remained for further analysis. The Q20 percentage was 96.13 %, and the GC percentage was 54.24 %. These clean reads were assembled into 101,597 contigs with a mean length of 378 bp. The N50 of contigs was 708 bp. These contigs were further assembled by paired-end joining and gap-filling, and clustered into unigenes. Finally, we obtained 72,667 unigenes, with a mean length of 537 bp. The N50 of unigenes was 747 bp. The size distribution indicated that the lengths of the 148 unigenes were more than 3,000 nt (Fig. 1). The contig and unigene size distributions were consistent, which indicated that the Illumina sequencing solution was reproducible and reliable.

**Table 1:** Output statistics of transcriptome sequencing

Total raw reads	61,733,120
Total clean reads	52,553,536
Total clean nucleotides (bp)	4,729,818,240
Q20 percentage	96.13 %
GC percentage	54.24 %
Total number of contigs	101,597
Total length of contigs (bp)	38,431,726
Mean length of contigs (bp)	378
N50 of contigs (bp)	708
Total number of unigenes (bp)	72,667
Total length of unigenes (bp)	39,029,083
Mean length of unigenes (bp)	537
N50 of unigenes (bp)	747

### Functional annotation and classification of unigenes

All of the unigenes were compared to the sequences in public databases, including NR, the Nucleic acid data bank (NT), Swiss-Prot, KEGG, COG, and GO database, using BLASTX with a cutoff e-value of 10^−5^ (Table 2). A total of 72,463 unigenes (99.68 % of all unigenes) returned a significant BLAST result.

**Table 2:** Statistics of annotation results

Item	NR	NT	Swiss-Prot	KEGG	COG	GO	Total
Total number of annotated unigenes	58,879	72,255	34,661	31,242	18,255	43,180	72,436
Percentage of annotated unigenes (%)	81.03	99.43	47.70	42.99	25.12	59.42	99.68

Unigenes were annotated with the databases of NR, NT, Swiss-Prot, KEGG, COG and GO. Then counted the number and percentage of unigenes annotated with each database

In order to annotate the transcriptome, a total of 72,667 unigenes were first examined against the NR database in NCBI using BLASTX with an E-value cut-off of 1e^−5^, which showed 58,879 (81.03 %) having significant BLAST hits (Table 2). The E-value distribution of significant hits revealed that 25,886 (43.97 %) of matched unigenes had strong homology (smaller than 1.0e-60), while the other homologous 32,993 (56.03 %) unigenes had E-values in the range of 1.0e-60 to 1.0e-5 (Additional file 5: Figure S5 (A)). The distribution of unigenes similarities presented that most of the BLASTX hits (48,515; 82.40 %) were higher than 95 %. Only 10,364 (17.60 %) of hits had sequence similarity values less than 95 % (Additional file 5: Figure S5 (B)). The species distribution revealed that 35,404 (60.13 %) unigenes had homology to rice (*Oryza sativa* L. ssp. *japonica*) sequences. Only 20,544 (34.89 %) unigenes had strong homology to rice (*Oryza sativa* L. ssp. *indica*) sequences (Additional file 5: Figure S5 (C)).

**Fig. 2:**
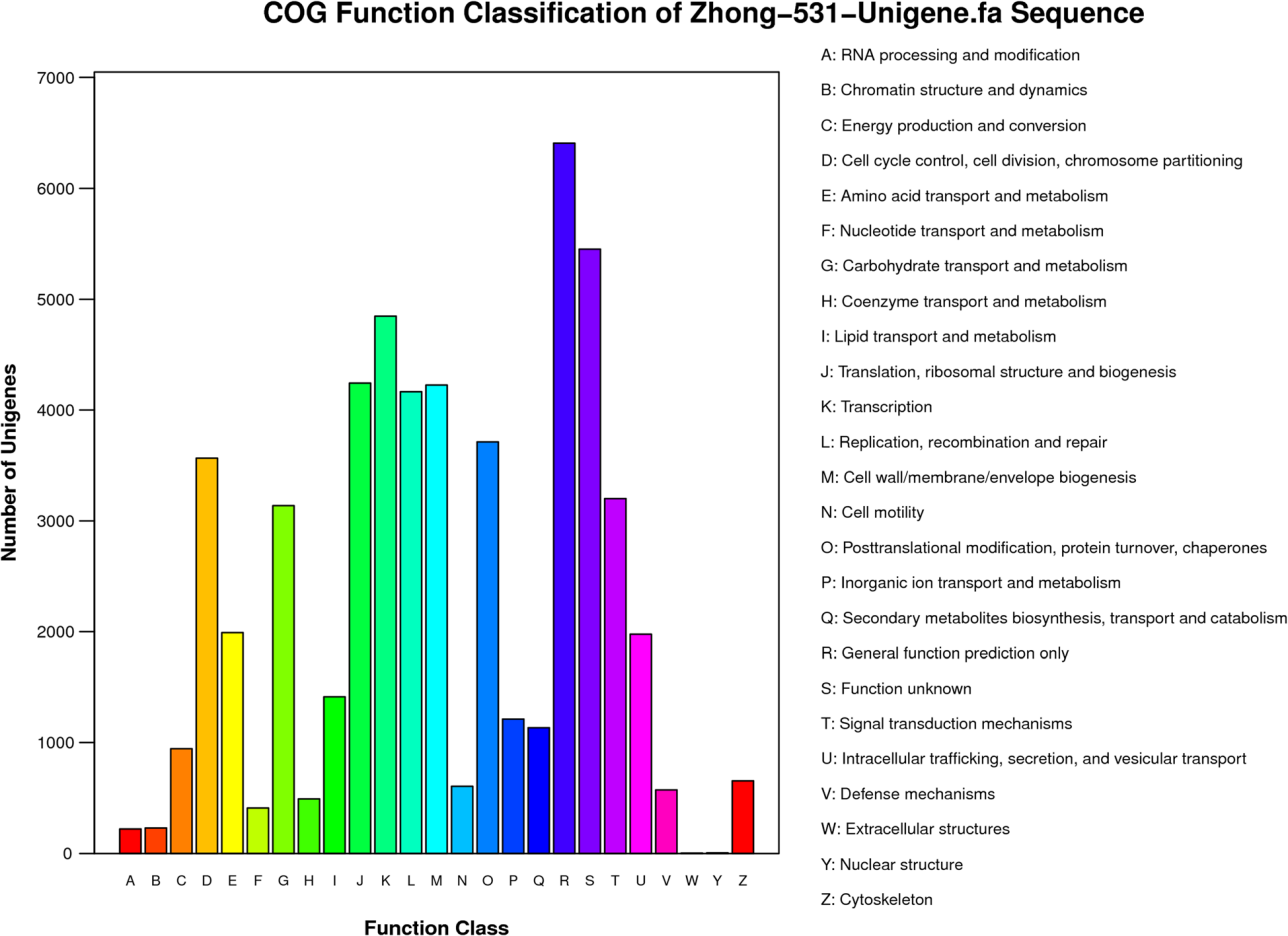
COG function classification of unigenes. The horizontal coordinates are function classes of COG, and the vertical coordinates are numbers of unigenes in one class. The notation on the right is the Full name of the functions in X-axis

The assembled unigenes were compared against the COG database to phylogenetically analyze widespread domain families. The results revealed 18,255 unigenes and 54,811 sequences with significant homology and assigned them to the appropriate COG clusters. These COG classifications were grouped into 25 functional categories (Fig. 2). Among these COG categories, the cluster “general function prediction only” (6,408; 11.69 %) represented the largest group, followed by “function unknown” (5,451; 9.59 %); “transcription” (4,848; 8.84 %); “translation, ribosomal structure and biogenesis” (4,243; 7.74 %); “cell wall/membrane/envelope biogenesis” (4,225; 7.71 %); “replication, recombination, and repair” (4,165; 7.60 %); “posttranslational modification, protein turnover, chaperones” (3,711; 6.77 %); “cell cycle control, cell division, chromosome partitioning” (3,564; 6.50 %); “signal transduction mechanisms” (3,202;5.84 %); and “carbohydrate transport and metabolism” (3,138; 5.73 %).

**Fig. 3:**
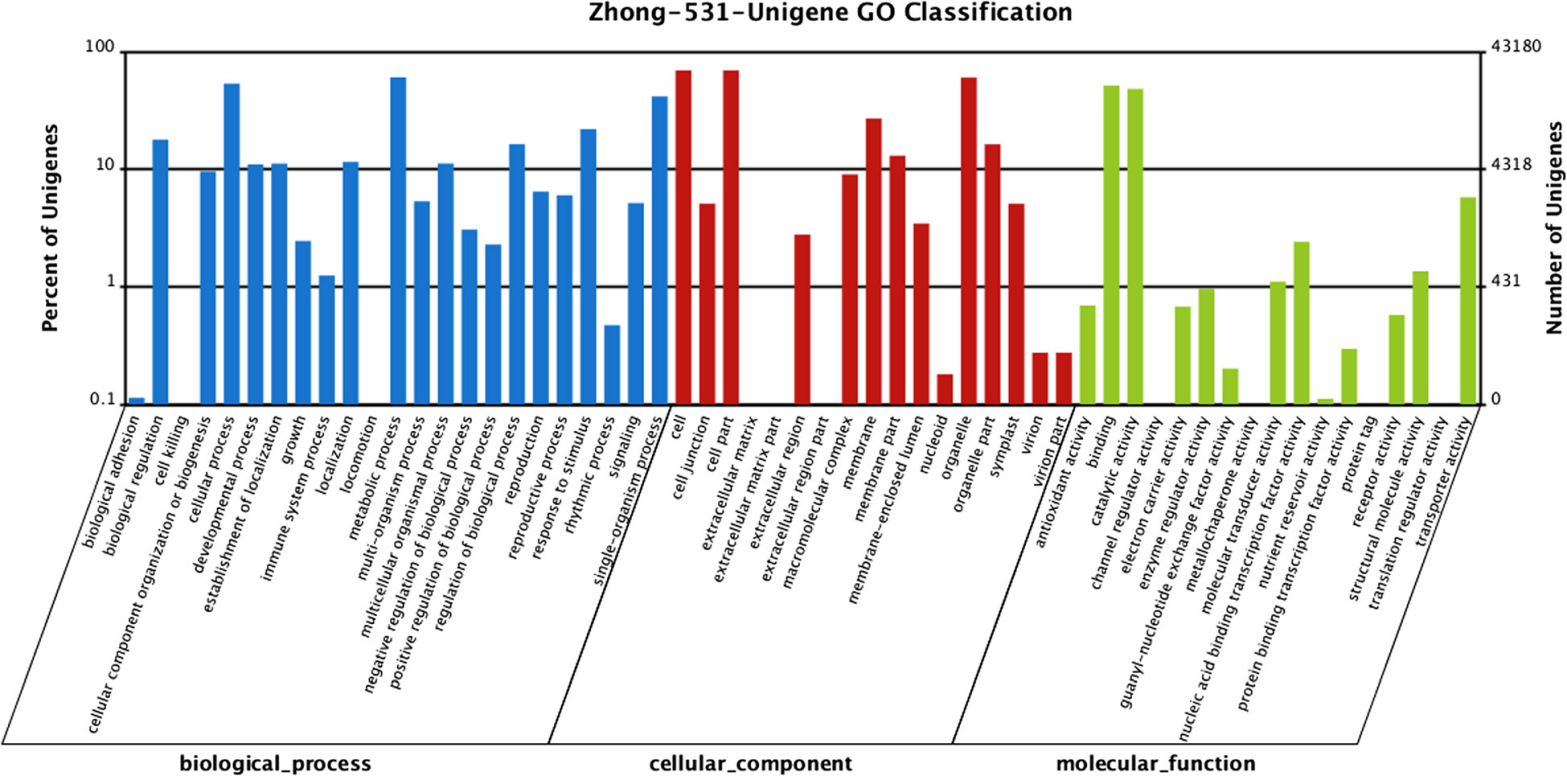
GO classification analysis of unigenes. Unigenes were annotated in three main categories: biological process, cellular component, and molecular function. The left y-axis indicates the percentage of a specific category of unigenes in that main category. The right y-axis indicates the number of unigenes in a category

GO assignments were used to classify the functions of the predicted unigenes. Based on sequence homology, 43,180 unigenes and 299,435 sequences could be categorized into three main categories with a total of 57 functional groups (Fig. 3). In each of the three main categories (biological process, cellular component, and molecular function) of the GO classification, “metabolic process”, “cell”, and “binding” were dominant. We also noticed a high-percentage of genes in the categories of “cellular process”, “cell part”, and “catalytic activity”.

The unigenes were further annotated by the KEGG database. In total, 31,242 unigenes were assigned to 128 KEGG pathways (Table 3). The pathway in which unigenes were most enriched was “metabolic pathways” (8,736), followed by “RNA transport” (4,026), “mRNA surveillance pathway” (3,569), and “Endocytosis” (3,145).

**Table 3:** KEGG pathway classification of transcriptome (the top 10)

Number	Pathway	All genes with pathway annotation (31,242)	Pathway ID
1	Metabolic pathways	8,736 (27.96 %)	ko01100
2	RNA transport	4,026 (12.89 %)	ko03013
3	mRNA surveillance pathway	3,569 (11.42 %)	ko03015
4	Endocytosis	3,145 (10.07 %)	ko04144
5	Glycerophospholipid metabolism	3,115 (9.97 %)	ko00564
6	Biosynthesis of secondary metabolites	3,074 (9.84 %)	ko01110
7	Ether lipid metabolism	2,924 (9.36 %)	ko00565
8	Plant-pathogen interaction	1,760 (5.63 %)	ko04626
9	Spliceosome	1,405 (4.5 %)	ko03040
10	Plant hormone signal transduction	1,365 (4.37 %)	ko04075

Note: The percentage of annotated unigenes in a pathway to the total number of unigenes is commented the small brackets

### Digital gene expression library sequencing (DGE sequencing)

Four DGE profiling libraries (NH, NN, HH, HN) were sequenced, which were tested by Agilent 2100 for quality control (Additional file 6: Table S1), and generated approximately 11 to 13 million high-quality reads for each library (Table 4). All samples had a RNA integrity number (RIN) and 28S:18S value of more than 8.0 and 1.6, respectively. To reveal the molecular events behind DGE profiles, we mapped the sequences of the DGE libraries to our transcriptome reference database generated in the above mentioned Illumina sequencing. The summary of reads mapped to reference genes (*de novo* assembly of transcriptome) (Table 4) was similar to the summary of reads mapped to reference genome (*Indica* rice database) (Additional file 7: Table S2). The percentage of clean reads among the raw reads in each library was above 99 % (Additional file 8: Figure S6). Among the clean reads, the number of sequences that could be mapped to unigenes ranged from 10 to 11 million, and the percentage of clean reads was beyond 84 % in the four DGE libraries. The vast majority of these mapped reads were uniquely matched to unigenes (>71 %), and the percentage of multi-position matched reads was less than 14 % (Table 4).

**Table 4:** Summary of reads mapped to reference genes (*de novo* assembly of transcriptome)

Samples	Total reads	Total base pairs	mapped reads	Perfect match	<=2 bp Mismatch	Unique match	Multi-position match	Total unmapped reads
ZHH-1	12,532,019 (100.00 %)	614,068,931 (100.00 %)	10,688,571 (85.29 %)	9,100,428 (72.62 %)	1,588,143 (12.67 %)	9,065,210 (72.34 %)	1,623,361 (12.95 %)	1,843,448 (14.71 %)
ZHH-2	11,933,716 (100.00 %)	584,752,084 (100.00 %)	10,130,167 (84.89 %)	8,669,541 (72.65 %)	1,460,626 (12.24 %)	8,601,124 (72.07 %)	1,529,043 (12.81 %)	1,803,549 (15.11 %)
ZHN-1	12,029,191 (100.00 %)	589,430,359 (100.00 %)	10,194,826 (84.75 %)	8,731,597 (72.59 %)	1,463,229 (12.16 %)	8,703,059 (72.35 %)	1,491,767 (12.40 %)	1,834,365 (15.25 %)
ZHN-2	12,381,854 (100.00 %)	606,710,846 (100.00 %)	10,448,097 (84.38 %)	8,879,274 (71.71 %)	1,568,823 (12.67 %)	8,891,358 (71.81 %)	1,556,739 (12.57 %)	1,933,757 (15.62 %)
ZNH-1	12,404,809 (100.00 %)	607,835,641 (100.00 %)	10,518,064 (84.79 %)	9,018,788 (72.70 %)	1,499,276 (12.09 %)	8,850,183 (71.34 %)	1,667,881 (13.45 %)	1,886,745 (15.21 %)
ZNH-2	12,057,086 (100.00 %)	590,797,214 (100.00 %)	10,235,897 (84.90 %)	8,758,708 (72.64 %)	1,477,189 (12.25 %)	8,619,541 (71.49 %)	1,616,356 (13.41 %)	1,821,189 (15.10 %)
ZNN-1	12,245,417 (100.00 %)	600,025,433 (100.00 %)	10,454,895 (85.38 %)	8,914,987 (72.80 %)	1,539,908 (12.58 %)	8,797,125 (71.84 %)	1,657,770 (13.54 %)	1,790,522 (14.62 %)
ZNN-2	11,936,315 (100.00 %)	584,879,435 (100.00 %)	10,150,654 (85.04 %)	8,632,637 (72.32 %)	1,518,017 (12.72 %)	8,601,628 (72.06 %)	1,549,026 (12.98 %)	1,785,661 (14.96 %)

Note: NH, Normal nitrogen level and high temperature treatment; NN, Normal nitrogen level and natural temperature treatment; HH, High nitrogen level and high temperature treatment; HN, High nitrogen level and natural temperature treatment. “Z” represents Zhong 531, 4 treatment combinations and 2 pooling duplicates. The same as follows

### Assessment of DGE sequencing

Sequence saturation analysis is used to measure the sequencing data of a sample. With the number of reads increasing, the number of detected genes is increasing. However, when the number of reads reaches a certain amount, the growth curve of detected genes flattens, which indicates that the number of detected genes has a tendency to saturation. As the Additional file 9: Figure S7 shows, when the sequencing amount of the four DGE libraries reached nearly 10 M, the number of detected genes almost ceased to increase.

During the RNA-Seq experiment, mRNA were first broken into short segments by chemical method and then sequenced. If the randomness is poor, reads preference to specific gene region will directly affect subsequent bioinformatics analysis. We used the distribution of reads on the reference genes to evaluate the randomness. Since reference genes have different lengths, the reads position on gene is standardized to a relative position (which is calculated as the ratio between reads location position on the gene and gene length), and then the number of reads in each position is counted. Additional file 10: Figure S8 was the result showing the distribution of reads on the reference genes of all four samples, which indicated that the randomness was good, and the reads in every position were evenly distributed.

Gene coverage is the percentage of a gene covered by reads. This value is determined as the ratio of the base number in a gene covered by unique mapping reads to the total bases number of that gene. The percentage of gene coverage above 90 % for all samples was about 40 % (Additional file 11: Figure S9). The expression level of each gene is determined by the numbers of reads uniquely mapped to the specific gene and the total number of uniquely mapped reads in the sample (RPKM). The summary results of gene expression and related information for all examples were given in Additional file 12: Table S3.

Good performance of screening group differentially expression genes needs a high correlation among the same replicates. So we used Pearson method to get coefficient of all genes between every two samples just for reference. The coefficient of all genes between every two samples was about 90 %, which indicated the performance of two replicates was excellent (Additional file 13: Figure S10).

### Differentially expressed genes (DEGs) among all samples

**Fig. 4:**
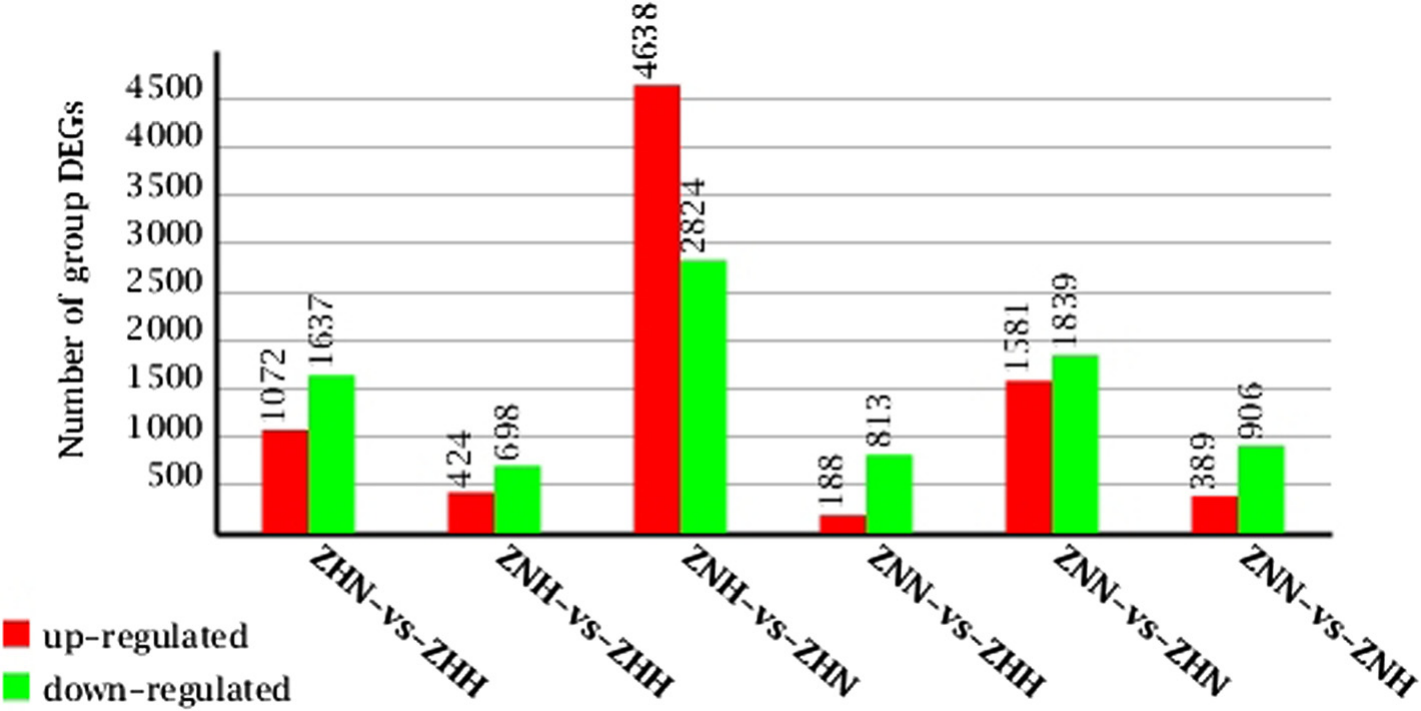
Stat chart of group differentially expressed genes. The differentially expressed genes were identified with the criteria (twofold or more change and *p* < 0.001)

The differentially expressed genes (DEGs) were identified in different samples. The following significant DEGs were identified: (a) between samples ZHN and ZHH, 1,072 and 1,637 genes were up- and down-regulated, respectively; (b) between samples ZNH and ZHH, 424 and 698 genes were up- and down-regulated, respectively; (c) between samples ZNH and ZHN, 4,638 and 2,824 genes were up- and down-regulated, respectively; (d) between samples ZNN and ZHH, 188 and 813 genes were up- and down-regulated, respectively; (e) between samples ZNN and ZHN, 1,581 and 1,839 genes were up- and down-regulated, respectively; (f) between samples ZNN and ZNH, 389 and 906 genes were up- and down-regulated, respectively (Fig. 4, Additional file 14: Table S4). In these DEGs, we found 151 and 323 DEGs temperature-responsive between ZNN and ZNH, and between ZHN and ZHH, respectively (Additional file 15: Table S5). These genes were principally associated with calcium-dependent protein kinase, cytochrome P450, flavonoid, heat shock protein, peroxidase, ubiquitin, photosynthesis, chlorophyll biosynthetic process, zinc transporter, transcription factor, sporopollenin biosynthetic process, and pollen exine formation and so on (Additional file 15: Table S5). Meanwhile, 203 and 144 DEGs in response to nitrogen were focused between ZNN and ZHN, and between ZNH and ZHH, respectively (Additional file 15: Table S5). These genes were principally related to glutamine synthetase, transcription factor, anthocyanin, amino acid transporter, leucine zipper protein, and hormone and so on (Additional file 15: Table S5).

**Fig. 5:**
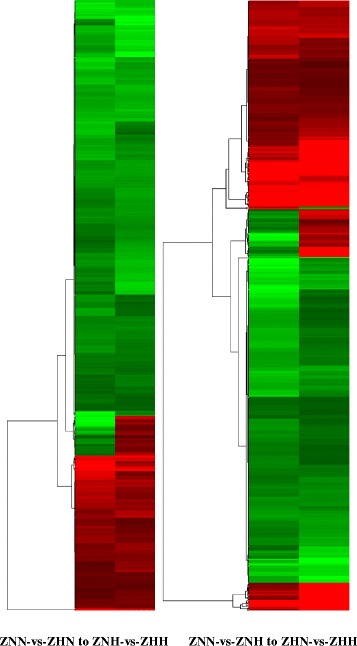
Hierarchical clustering of differentially expressed genes. Each column represents an experimental condition, each row represents a gene. For differentially expression genes, its log_2_ (RPKM) will be clustered, and red means up regulation and green means down regulation, and the color is more close to red or green, the more highly this gene expresses

In order to further illuminate the effects of temperature and nitrogen, we compare the DEGs in different samples. A total of 532 DEGs occurred simultaneously in the two comparisons (ZNN-vs-ZNH and ZHN-vs-ZHH), and 671 DEGs were co-expressed in ZNN-vs-ZNH and ZHN-vs-ZHH, respectively (Additional file 16: Table S6). A total of 114 and 111 DEGs associated with temperature and nitrogen were screened out (Additional file 17: Table S7), and the expression pattern analysis of DEGs under the same nitrogen level and the same conditions of temperature was clustered in Fig. 5, respectively.

To understand the functions of these differentially expressed genes, all the DEGs were mapped to terms in the GO database and compared to the whole transcriptome background. The DEGs had a GO ID and can be categorized into small functional groups in three main categories (biological process, cellular component, and molecular function) of the GO classification (Additional file 18: Table S8, Additional file 19: Figure S11). Based on sequence homology, the following significant DEGs annotated by the GO database were identified: (a) in ZNN-vs-ZNH and ZHN-vs-ZHH, 50 and 51 functional groups were categorized, respectively; (b) in ZNN-vs-ZHN and ZNH-vs-ZHH, 55 and 44 functional groups were categorized, respectively. Among these groups, “metabolic process” and “cellular process” were dominant within the “biological process” category, the “cell” and “cell part” categories were dominant in the “cellular component” category, and “catalytic activity” and “binding” were dominant in the “molecular function” category (Additional file 19: Figure S11).

**Fig. 6:**
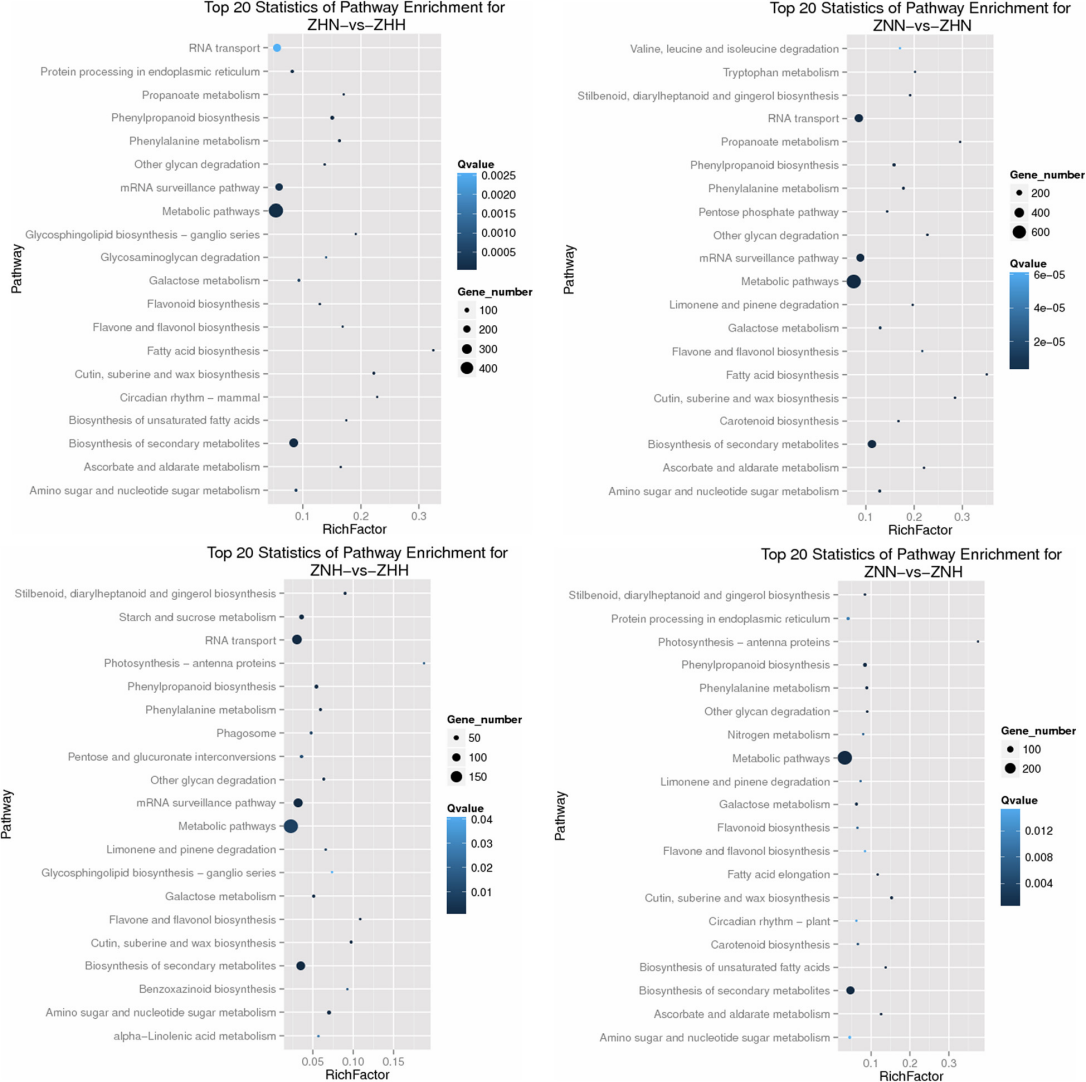
Scatter plot of KEGG pathway enrichment statistics. RichFactor is the ratio of differentially expressed gene numbers annotated in this pathway term to all gene numbers annotated in this pathway term. Greater RichFactor means greater intensiveness. Q-value is corrected P-value ranging from 0 ~ 1, and its less value means greater intensiveness. We just display the top 20 pathway terms enriched by KEGG database

To further investigate the biochemical pathways of these DEGs, we mapped all of the DEGs to terms in KEGG database and compared this with the whole transcriptome background. The DEGs had a KO ID and could be categorized into small pathways (Additional file 20: Table S9). For ZNN-vs-ZNH and ZHN-vs-ZHH, of the 1,295 and 2,709 DEGs, 716 and 1,410 unigenes had a KO ID and could be categorized into 95 and 109 pathways, respectively. Of those, 26 pathways were significantly enriched (Q value < 0.05), and genes involved in metabolic pathways were the most significantly enriched (Additional file 20: Table S9). For ZNN-vs-ZHN and ZNH-vs-ZHH, of the 3,420 and 1,122 DEGs, 1,902 and 584 unigenes had a KO ID and could be categorized into 116 and 93 pathways, respectively. Of those, 35 and 20 pathways were significantly enriched (Q value < 0.05), and genes involved in metabolic pathways were the most significantly enriched, respectively (Additional file 20: Table S9). The top 20 KEGG pathways of DEGs in these four comparisons mentioned above are showed in Fig. 6.

### Effects of nitrogen level and high temperature at late spikelet differentiation stage (meiosis) on spikelets fertility of rice

**Fig. 7:**
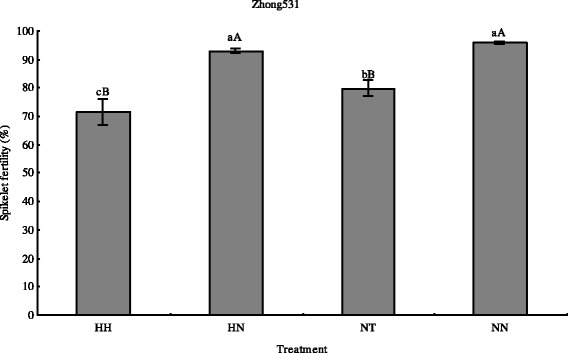
Effects of nitrogen level and high temperature on spikelet fertility of rice. Data show as mean ± standard error. Bars marked with the same letters indicate no significant difference at 5 % level or 1 % level. NH, Normal nitrogen level and high temperature treatment; NN, Normal nitrogen level and natural temperature treatment; HH, High nitrogen level and high temperature treatment; HN, High nitrogen level and natural temperature treatment

To test the effects of high nitrogen level and high temperature, we investigated the spikelet fertility in the four combinations (Fig. 7). High temperature at meiosis stage significantly decreased spikelet fertility of Zhong531 compared with natural temperature at high nitrogen level or normal nitrogen level (*p* < 0.01). Under high temperature or natural temperature, spikelet fertility decreased at high nitrogen level compared with normal nitrogen level, and the difference under high temperature was significantly (*p* < 0.05). These results imply that excessive high nitrogen level contributed to increase the effects of high temperature on spikelet fertility.

## Discussion

In recent years, RNA-seq has been used as a powerful and cost-efficient tool for mining gene resources and functions. In this study, we performed *de novo* assembly of transcriptome in rice spikelet using the platform of Illumina Hiseq 2000, and obtained a total 4.73 Gb of transcriptome data. In the results of assembly, 72,667 unigenes were detected, with the total length 39,029,083 nt, average length 537 nt, and N50 747 nt. For functional annotation analysis, unigenes were annotated with the databases of NR (58,879), NT (72,255), Swiss-Prot (34,661), KEGG (31,242), COG (18,255), and GO (43,180) by using BLASTX with a cutoff e-value of 10^−5^, respectively, and the total annotated unigenes were 72,436. We obtained over 10 million clean reads from the samples of four digital gene expression profiling libraries (NH, NN, HH, HN), respectively. A lot of up-regulated and down-regulated genes were differentially expressed among the four libraries. The DEGs data provided comprehensive information of the gene expression in rice spikelet, facilitating our understanding of the molecular mechanisms of the different physiological aspects of rice spikelet in response to temperature interacting with nitrogen at meiosis stage.

Rice growth is challenged by fluctuations in environmental factors specially temperature on almost daily basis. To minimize high temperature damage, rice has developed adaptations and heat tolerance during evolution through the regulation of gene expression and changes in cellular processes. It’s well known that heat shock proteins (HSPs) are responsive to heat stress in plants [53–55]. In plants, HSPs are encoded by nuclear multigene families and localized in different cellular compartments. HSPs generally function as molecular chaperones, and are divided into HSP100, HSP90, HSP70, HSP60, HSP40 and HSP20 or small heat shock proteins (sHSPs) [56, 57]. When plants are exposed to elevated temperature, the protective HSPs have increased levels of protein and gene expression [58–61]. *AtHSP70-15-*deficient plants under heat stress resulted in drastically increase in mortality, indicating that *AtHSP70-15* played an essential role during normal growth and in the heat response of *Arabidopsis* [62]. A heat-tolerant rice N22 with 71 % spikelet fertility had a cold (putative) and a heat (unknown) shock protein significantly up-regulated under 38 °C during anthesis, indicating that the shock proteins may have a greater contribution to the heat tolerance for N22 [5]. Our results revealed that HSP90, HSP70 and HSP20 in these differentially expressed genes (DEGs) were positively responsive to high temperature (Additional file 15: Table S5). Thus, the Hsps genes play an important part in response to heat stress in rice spikelet. Moreover, there were 19 and 26 DEGs annotated as HSPs in between ZNN-vs-ZNH and ZHN-vs-ZHH (Additional file 15: Table S5), respectively, and 18 HSPs occurred simultaneously in the two comparisons (Additional file 17: Table S7). In these DEGs, the gene expression of *CL3468.Contig1*, *Unigene9201*, *Unigene5315*, *Unigene5314*, and *Unigene25510* in ZNN-vs-ZNH was extremely significant with log_2_Ratio ≥ 4.0, and *CL3468.Contig1*, *Unigene5315*, *Unigene5314* in ZHN-vs-ZHH was extremely significant with log_2_Ratio ≥ 5.0. Manwhile, the log_2_Ratio value of *Unigene9201*, *Unigene47969*, *Unigene24821*, *Unigene24300*, *Unigene41142*, *Unigene41141*, *Unigene41143*, and *Unigene25395* of ZHN-vs-ZHH was greater compared to ZNN-vs-ZNH, and the gene expression of other 7 HSP genes was on the contrary. These results may explain the gene expression difference of rice spikelet in response to high temperature under two different nitrogen levels.

The cytochrome P450, ubiquitin protein, flavonoid, and transcription factor genes have been extensively studied in plants in response to heat stresses [12, 63, 64]. Most of the heat-responsive (P450 family) genes were repressed to some extent, and only 10 P450 family genes were up-regulated under 40 °C at the stage 8 of anther development [12]. High temperature reduced spikelet fertility of rice, DNA microarray analysis revealed that cytochrome P450 family genes were designated as high temperature-repressed genes in the anther at the microspore stage [11]. In this study, we found that 17 cytochrome P450 genes were all down-regulated in ZNN-vs-ZNH, but 14 and 8 cytochrome P450 genes were up-regulated and down-regulated in ZHN-vs-ZHH, respectively (Additional file 15: Table S5). Furthermore, 6 co-expressed cytochrome P450 genes (*Unigene7325*, *Unigene42477*, *Unigene42476*, *Unigene45111*, *CL2270*.Contig1, and *Unigene42480*) showed different expression in two comparisons with all genes down-regulated in ZNN-vs-ZNH and 3 genes up-regulated in ZHN-vs-ZHH (Additional file 17: Table S7). Besides, the genes belonging to ubiquitin protein, flavonoid, peroxidase, and transcription factor also showed expression differences between ZNN-vs-ZNH and ZHN-vs-ZHH.

Nitrogen is one of the major macronutrients for higher plants and can be served as a signal molecule to regulate plant development, physiology, and metabolism [14, 65]. Peng et al. [25] found that nitrogen limitation altered the expression levels of 629 genes with 340 of them up-regulated and 289 of them down-regulated in *Arabidopsis*. The up-regulated group included the genes involved in protein degradation and the biosynthesis of anthocyanin, while the down-regulated group contained the genes functioning in photosynthesis and the synthesis of nitrogenous macromolecules such as chlorophyll, proteins, amino acids and nucleotides. Plant hormone play many biological roles in plants. Hirano et al. [66] analyzed the global expression profiles of genes related to phytohormones in microspore/pollen (MS/POL) development. The genes required for IAA and gibberellin (GA) synthesis were coordinately expressed during the later MS/POL developmental stage. In contrast, genes for GA signaling were preferentially expressed during the early stage. Phenotypic analysis revealed that the GA-deficient mutant reduced pollen elongation was defective in pollen tube elongation, resulting in a low spikelet fertilization frequency, whereas the GA-insensitive semidominant mutant was mainly defective in viable pollen production. Furthermore, the GA biosynthesis genes were preferentially expressed after meiosis during pollen development [67]. It was reported that reduction of gibberellin by low temperature (approximately 19 °C) disrupted pollen development and caused severe reduction of seed setting in rice [68]. It was reported that nitrogen responsive genes encoded transcription factors, signal transduction process, and proteins required for plant hormone synthesis and response [69–71]. In this study, we found 203 and 144 nitrogen-responsive DEGs in ZNN-vs-ZHN and ZNH-vs-ZHH, respectively (Additional file 15: Table S5). Numerous nitrogen responsive genes encoded plant hormone synthesis and response such as abscisic acid, auxin, gibberellin, cytokinin, and ethylene. Furthermore, a total of 111 DEGs associated with nitrogen co-expressed (Additional file 17: Table S7). WRKY genes encode transcription factors with a WRKY domain that belongs to zinc-finger proteins, which play important roles in responses to abiotic stress in rice [72, 73]. In these co-expressed DEGs, 8 WRKY transcription factor genes were all down-regulated, and the log_2_Ratio value of *Unigene20481*, *CL4288.Contig1*, *CL554.Contig1*, *CL4639.Contig1*, *CL1348.Contig1*, and *Unigene16792* of ZNN-vs-ZHN was greater than that of ZNH-vs-ZHH. High temperature cause comprehensive alterations in transcription, but application of auxin can block the transcriptional alterations, leading to the production of normal pollen grains, and the normal seed setting rate under increasing temperatures [74]. We found that 9 auxin-regulated genes with 4 down-regulated and 5 up-regulated DEGs were co-expressed. Our results revealed the gene expression difference of rice spikelet in response to nitrogen.

High temperature during flowering (anthesis and fertilization) greatly reduces spikelet fertility in rice [5]. Pollen viability in rice plants exposed to (39 °C) was lower than that in control plants, and the pollen grains were very poorly attached and displayed limited germination on the stigma under high temperature, leading to reduced spikelet fertility. Spikelet fertility is closely related to pollen exine formation, pollen tube growth, pollen germination, and pollen development in plants. An ABC transporter gene (OsABCG15) was identified to be involved in pollen development in rice. Wu et al. [75] showed that OsABCG15 played an essential role in the formation of the rice anther cuticle and pollen exine. Ling et al. [76] identified 291 mature anther-preferentially expressed genes (OsSTA) in rice, and OsSTA genes were associated with pollen fertility, pollen germination and anther dehiscence in rice. The *pollen semi-sterility1* (*PSS1*) encoded a kinesin-1-like protein to regulate anther dehiscence in rice [77]. It was pointed out that a RING-type E3 ubiquitin ligase, POLLEN TUBE BLOCKED 1 (PTB1), positively regulated the rice panicle seed setting rate by promoting pollen tube growth [78]. OsAP65, a rice aspartic protease, was essential for male fertility and played a important role in pollen germination and pollen tube growth [79]. Chueasiri et al. [80] quantified gene expression in anthers of temperature-sensitive rice plants grown in controlled growth rooms (26 °C and 32 °C) for fertile and sterile conditions, the results indicated that plant orosomucoids-like proteins influenced sphingolipid homeostasis, and deletion of this gene affected spikelet fertility resulting from abnormal pollen development. The transcription factor *bHLH142* was identified as a pivotal role in tapetal programmed cell death and pollen development during early meiosis in rice [81]. In our study, the expression profile revealed that 15 spikelet genes annotated to GO database including 3 pollen development, 9 pollen exine formation, 1 pollen germination, 1 pollen tube development, and 1 sporopollenin biosynthetic process (pollen exine formation) were all down-regulated in ZNN-vs-ZNH (Additional file 15: Table S5). Many genes including 4 microsporogenesis, 11 flower development, 13 pollen development, 3 pollen tube growth, 12 pollen germination, 12 pollen exine formation, 9 pollen wall assembly, and 14 sporopollenin biosynthetic process from GO annotation were identified as spikelet genes in response to high temperature interacting with high nitrogen level in ZHN-vs-ZHH (Additional file 15: Table S5). Moreover, we found that 8 pollen exine formation, and 8 sporopollenin biosynthetic process DEGs with all genes down-regulated were co-expressed. Interestingly, in these co-expressed DEGs, the log_2_Ratio absolute value of spikelet genes including 1 pollen development gene (*CL3271.Contig1*), 7 pollen exine formation genes (*Unigene45103*, *Unigene32014*, *Unigene32018*, *Unigene32017*, *Unigene42236*, *Unigene42231*, *Unigene42234*), and 5 sporopollenin biosynthetic process genes (*Unigene43852*, *Unigene32623*, *Unigene23940*, *Unigene23939*, *Unigene23941*) of ZNN-vs-ZNH was lower than that of ZHN-vs-ZHH (Additional file 17: Table S7). The co-expressed DEGs especially pollen exine formation and sporopollenin biosynthetic process genes may suggest they play important roles in rice spikelet in response to high temperature at high nitrogen level. Accordingly, high temperature at meiosis stage significantly decreased spikelet fertility, and spikelet fertility decreased more significantly under high temperature interacting with high nitrogen level (Fig. 7). Therefore, these genes may be good candidates for crop improvement. In addition, we found 151 DEGs with 40 up-regulated and 111 down-regulated genes, and 323 DEGs with 150 up-regulated and 173 down-regulated genes in response to high temperature were identified in ZNN-vs-ZNH and ZHN-vs-ZHH, respectively (Additional file 15: Table S5), which indicating more DEGs were related to high temperature at high nitrogen level.

## Conclusions

In summary, we performed a combination of RNA-seq and digital gene expression sequencing to identify the genes involved in rice spikelet treated by different temperatures and nitrogen levels. This study produced abundant data for research on the molecular mechanisms of the rice spikelet. We highlight the link between the pollen exine formation, sporopollenin biosynthetic process genes and the decreased spikelet fertility. To the best of our knowledge, the present study is the first to attempt to perform a *de novo* assembly of transcriptome sequencing of the temperature and nitrogen’s interactive effects in rice spikelet, which may extend our understanding of the complex molecular and cellular events in spikelet and facilitate identification of temperature and nitrogen’s interactive effects on plants.

### Availability of supporting data

RNA-seq data used in the present study have been deposited into the NCBI Sequence Read Archive (SRA, http://www.ncbi.nlm.nih.gov/sra/) under the accession number of SRP058792 (SRR2043067, SRR2043068, SRR2043070, SRR2043072). All the supporting data are included as additional files.

## Additional files

Additional file 1: Figure S1. The Pipeline of experiments before Illumine high sequencing. (PNG 14 kb) Additional file 2: Figure S2. The Pipeline of bioinformatics analysis of transcriptome. (JPEG 51 kb) Additional file 3: Figure S3. The assembly process of unigenes. (JPEG 134 kb) Additional file 4: Figure S4. The scheme showing standard bioinformatics analysis of RNA-Seq (Quantification). (PNG 36 kb) Additional file 5: Figure S5. The distribution of the result of NR annotation. (A) Figure of E-value distribution; (B) Figure of similarity (identity) distribution; (C) Figure of species distribution. (PNG 174 kb) Additional file 6: Table S1. The quality control of testing RNA using in digital gene expression (DGE) sequencing. (XLS 16 kb) Additional file 7: Table S2. The summary of mapping result (reads mapped to reference genome, *Indica* rice database). (XLS 17 kb) Additional file 8: Figure S6. The quality assessment of reads (DOC 131 kb) Additional file 9: Figure S7. The sequencing saturation analysis. (DOC 89 kb) Additional file 10: Figure S8. The randomness assessment. (DOC 94 kb) Additional file 11: Figure S9. The gene coverage. (DOC 228 kb) Additional file 12: Table S3. The quantification of gene expression. (XLS 31048 kb) Additional file 13: Figure S10. The correlation analysis of Pearson coefficient in gene expression between two pooling samples duplicates. (DOC 93 kb) Additional file 14: Table S4. The differentially expressed genes (DEGs) were identified in different samples. (XLS 4299 kb) Additional file 15: Table S5. The differentially expressed genes (DEGs) response to temperature, and nitrogen were focused in different samples. (XLS 145 kb) Additional file 16: Table S6. The summary of differentially expressed genes (DEGs) occurred simultaneously in different samples. (XLS 438 kb) Additional file 17: Table S7. The Summary of co-expressed differentially expressed genes (DEGs) in response to temperature and nitrogen in different samples. (XLS 49 kb) Additional file 18: Table S8. The Gene GO Ontology enrichment analysis of the differentially expressed genes (DEGs). (XLS 1686 kb) Additional file 19: Figure S11. The GO classification analysis of differentially expressed genes (DEGs). (DOC 693 kb) Additional file 20: Table S9. The KEGG pathway enrichment analysis of the differentially expressed genes (DEGs). (XLS 654 kb)
