# Supplementary figures and images for: RNA-seq reveals differentially expressed genes of rice (Oryza sativa) spikelet in response to temperature interacting with nitrogen at meiosis stage
Source: BMC Genomics. 2015 Nov 17;16:959. doi: 10.1186/s12864-015-2141-9 (PMC4650392; doi:10.1186/s12864-015-2141-9)

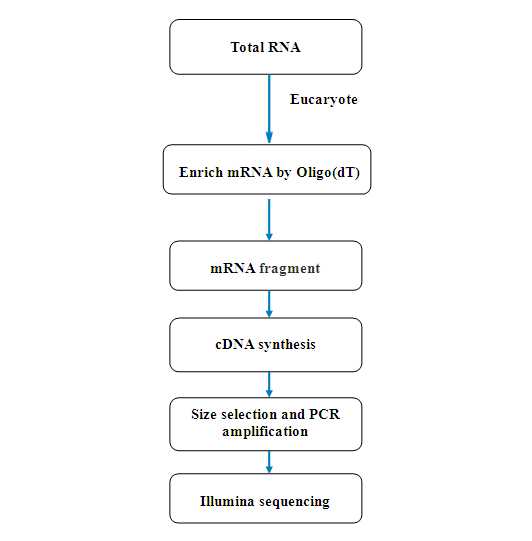

Supplement: Additional file 1: Figure S1. — The Pipeline of experiments before Illumine high sequencing. (PNG 14 kb) [file 12864_2015_2141_MOESM1_ESM.png]

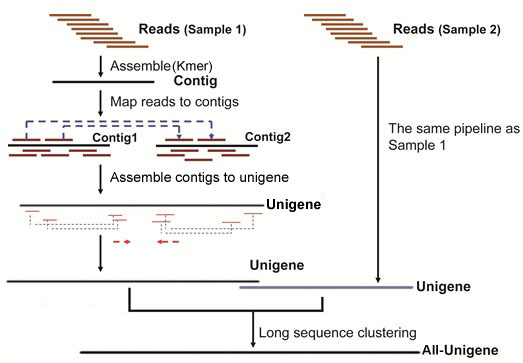

Supplement: Additional file 2: Figure S2. — The Pipeline of bioinformatics analysis of transcriptome. (JPEG 51 kb) [file 12864_2015_2141_MOESM2_ESM.jpg]

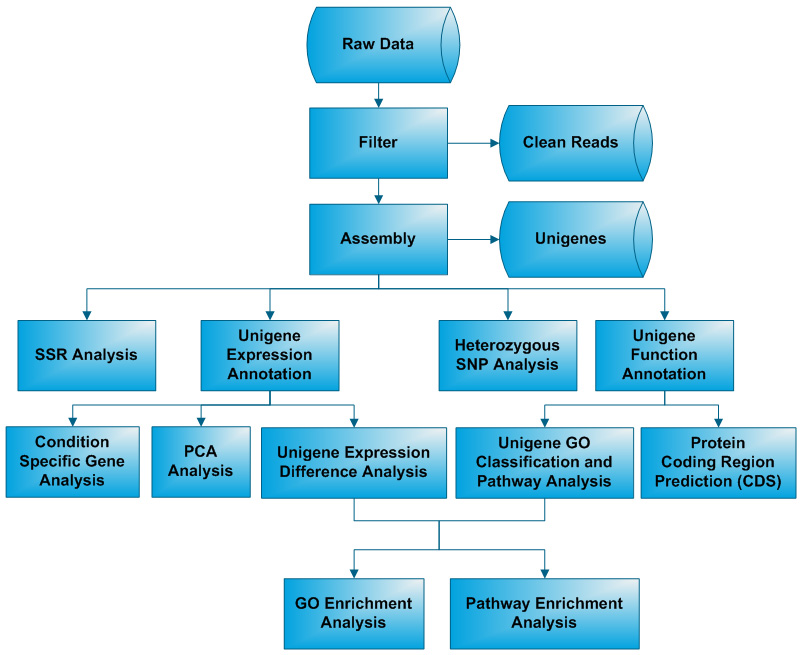

Supplement: Additional file 3: Figure S3. — The assembly process of unigenes. (JPEG 134 kb) [file 12864_2015_2141_MOESM3_ESM.jpg]

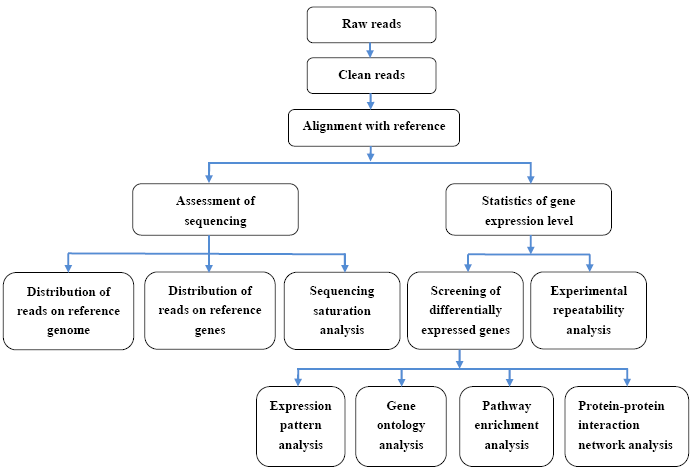

Supplement: Additional file 4: Figure S4. — The scheme showing standard bioinformatics analysis of RNA-Seq (Quantification). (PNG 36 kb) [file 12864_2015_2141_MOESM4_ESM.png]

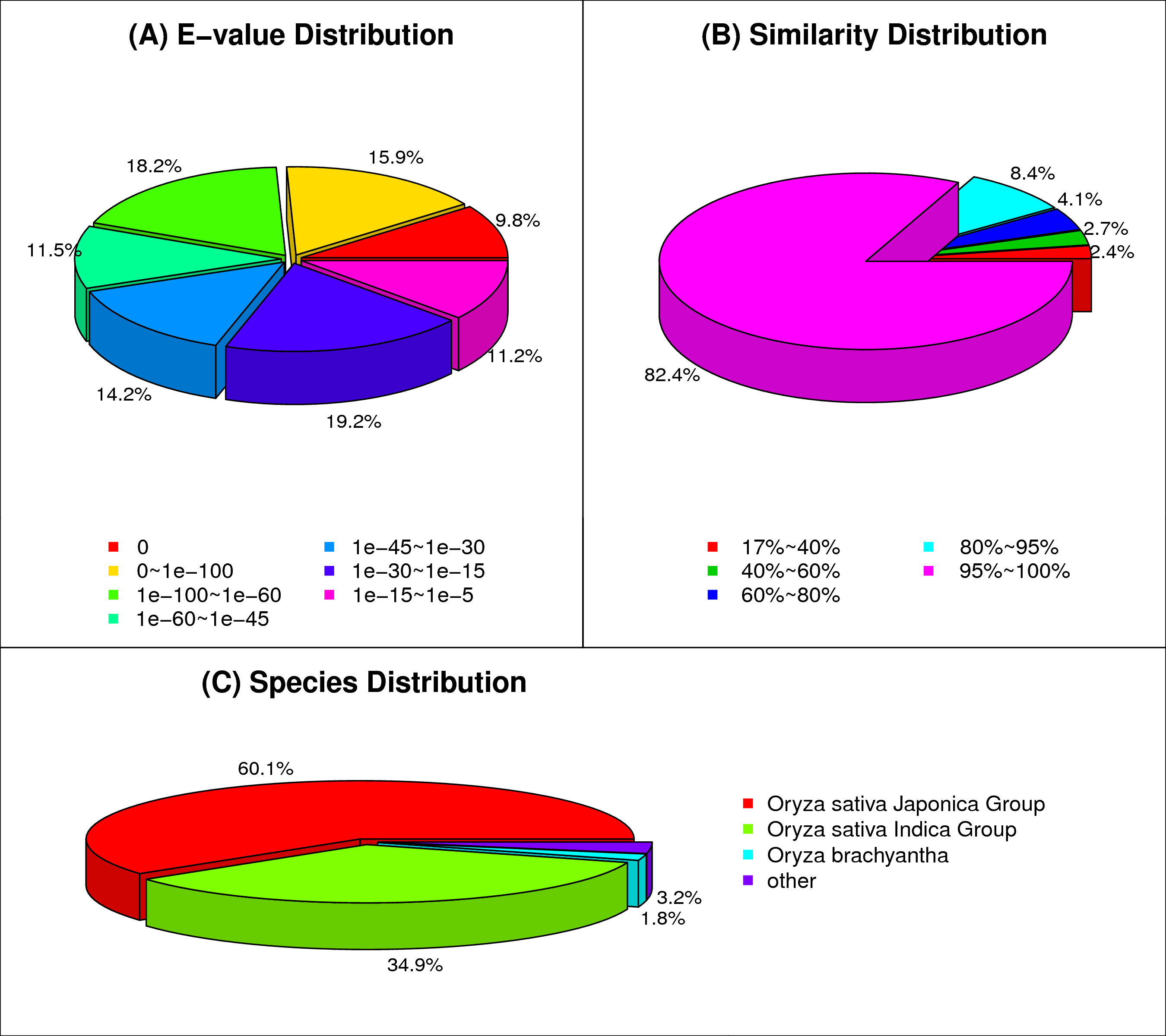

Supplement: Additional file 5: Figure S5. — The distribution of the result of NR annotation. (A) Figure of E-value distribution; (B) Figure of similarity (identity) distribution; (C) Figure of species distribution. (PNG 174 kb) [file 12864_2015_2141_MOESM5_ESM.png]

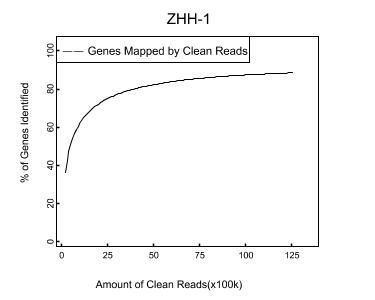

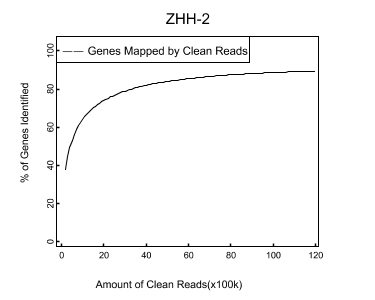

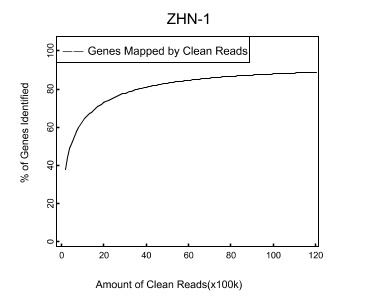

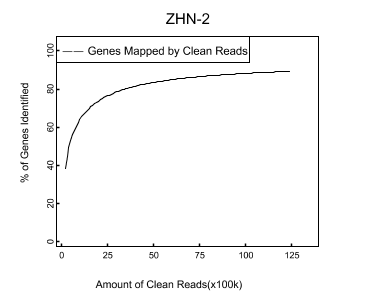

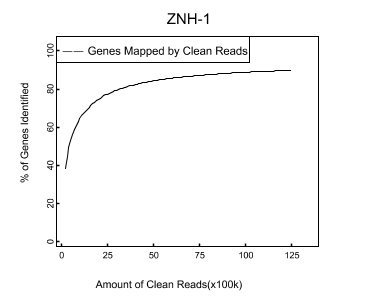

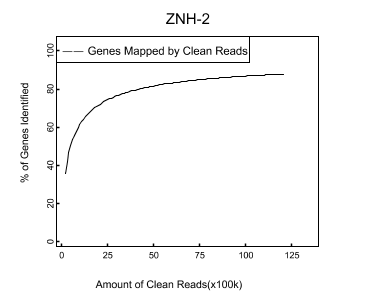

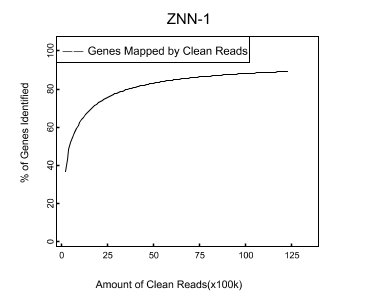

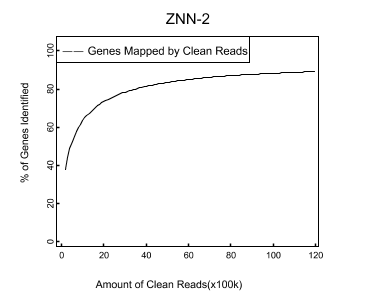


**Fig S7 Sequencing saturation analysis**

Supplement: Additional file 9: Figure S7. — The sequencing saturation analysis. (DOC 89 kb) [file 12864_2015_2141_MOESM9_ESM.doc]

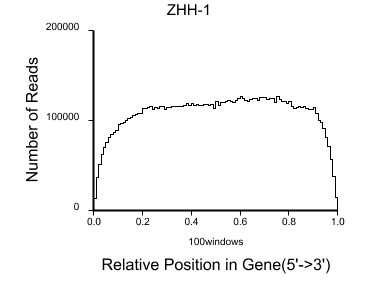

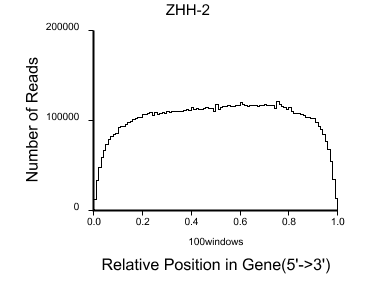

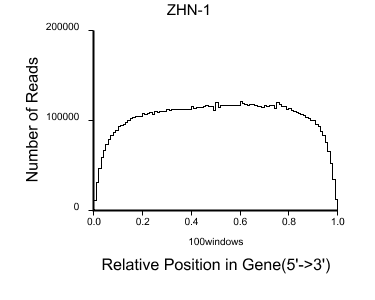

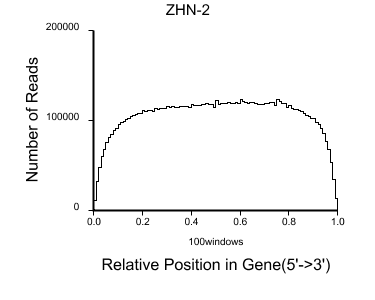

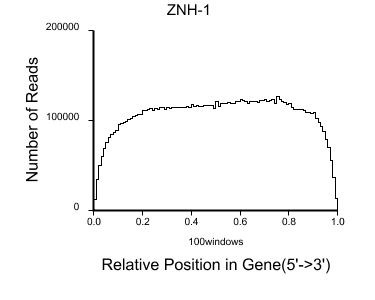

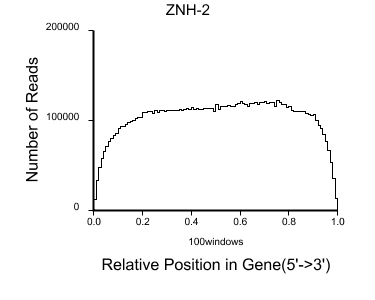


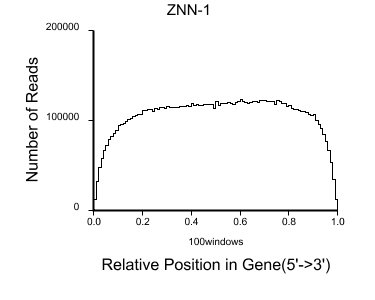

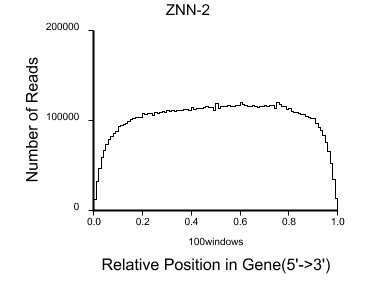
 **Fig S8 Randomness assessment**

Supplement: Additional file 10: Figure S8. — The randomness assessment. (DOC 94 kb) [file 12864_2015_2141_MOESM10_ESM.doc]

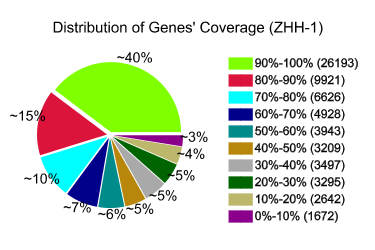

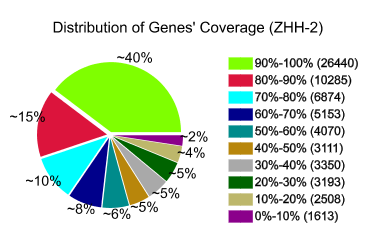

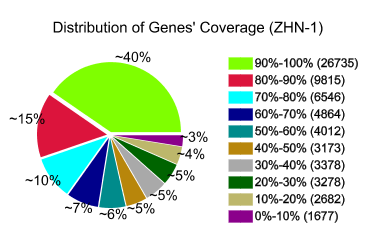

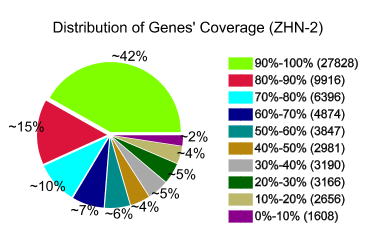

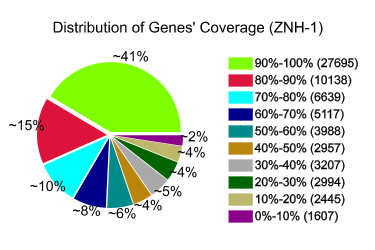

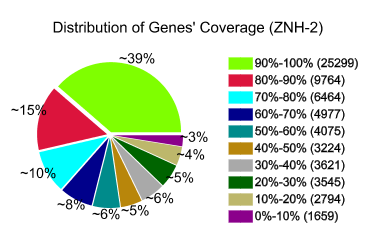

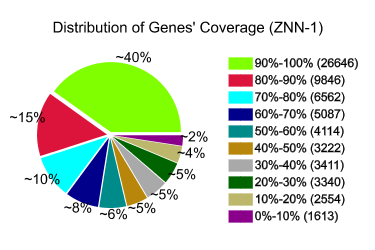

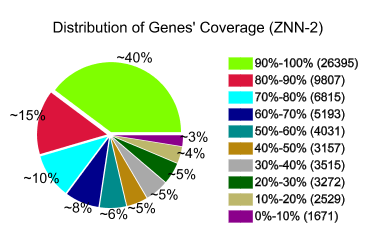


**Fig S9 Gene coverage**

Supplement: Additional file 11: Figure S9. — The gene coverage. (DOC 228 kb) [file 12864_2015_2141_MOESM11_ESM.doc]
