# Supplementary material for: RNA-seq reveals differentially expressed genes of rice (Oryza sativa) spikelet in response to temperature interacting with nitrogen at meiosis stage
Source: BMC Genomics. 2015 Nov 17;16:959. doi: 10.1186/s12864-015-2141-9 (PMC4650392; doi:10.1186/s12864-015-2141-9)
Supplement: Additional file 8: Figure S6. — The quality assessment of reads (DOC 131 kb) [file 12864_2015_2141_MOESM8_ESM.doc]

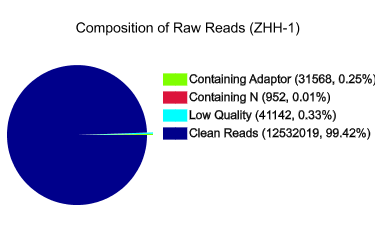

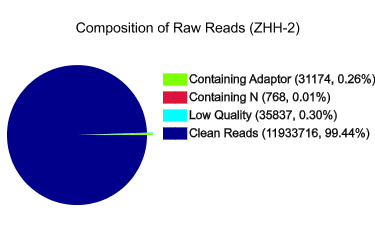


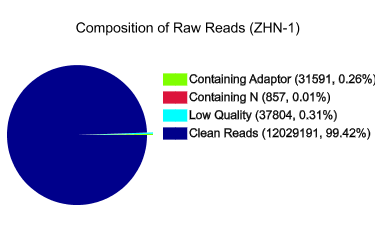

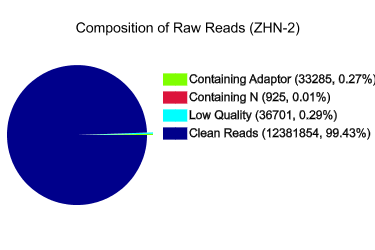

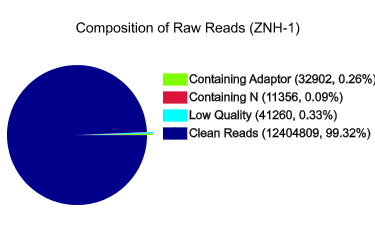

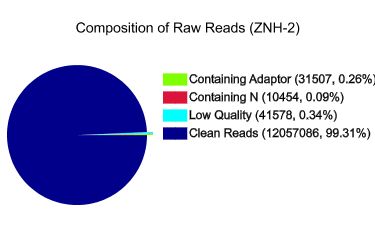

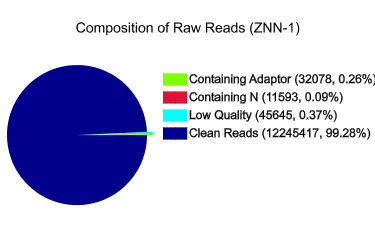

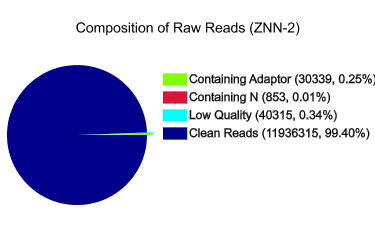


**Fig S6 Quality assessment of reads**

The numbers and percentage of reads containing adaptor, containing N, low quality reads, and clean reads, are shown.
