# Supplementary material for: RNA-seq reveals differentially expressed genes of rice (Oryza sativa) spikelet in response to temperature interacting with nitrogen at meiosis stage
Source: BMC Genomics. 2015 Nov 17;16:959. doi: 10.1186/s12864-015-2141-9 (PMC4650392; doi:10.1186/s12864-015-2141-9)
Supplement: Additional file 13: Figure S10. — The correlation analysis of Pearson coefficient in gene expression between two pooling samples duplicates. (DOC 93 kb) [file 12864_2015_2141_MOESM13_ESM.doc]

**
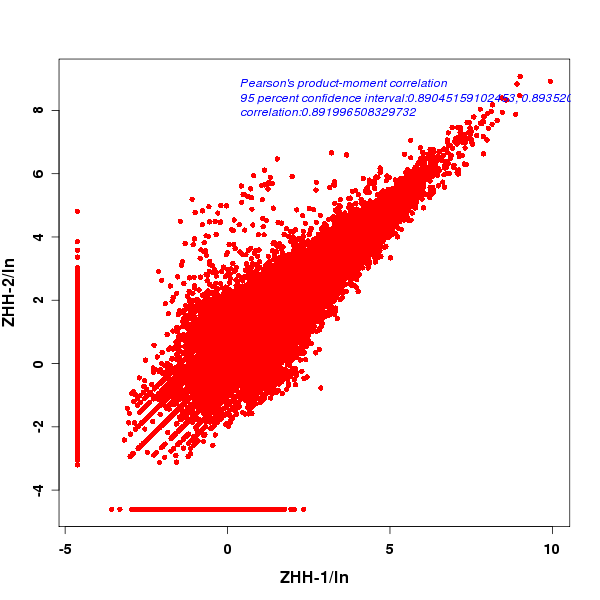

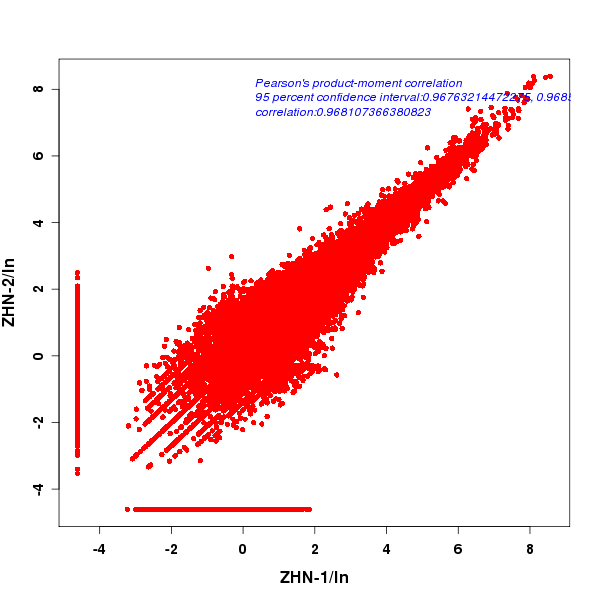

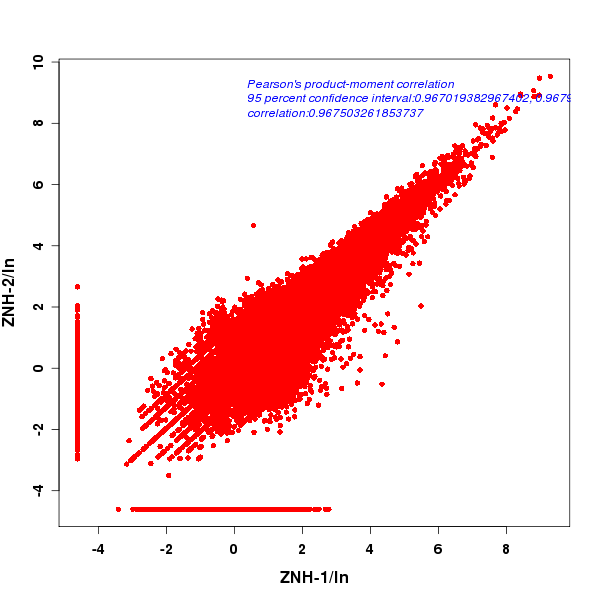

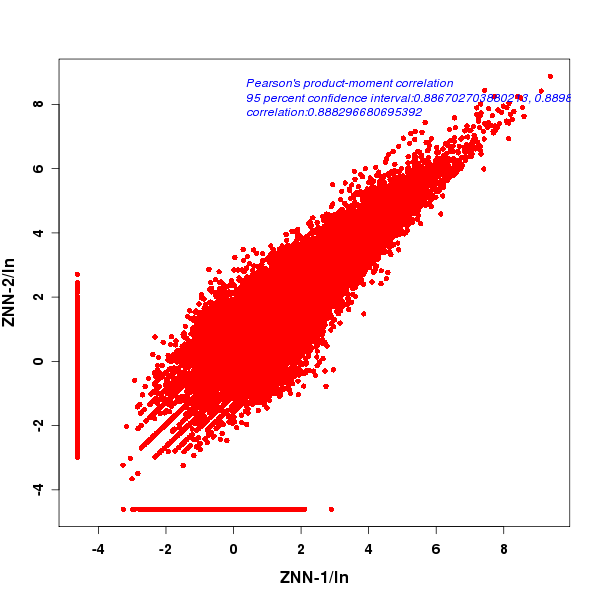
**

**Fig S10 Correlation analysis of Pearson coefficient in gene expression between two pooling samples duplicates**
