# Supplementary material for: RNA-seq reveals differentially expressed genes of rice (Oryza sativa) spikelet in response to temperature interacting with nitrogen at meiosis stage
Source: BMC Genomics. 2015 Nov 17;16:959. doi: 10.1186/s12864-015-2141-9 (PMC4650392; doi:10.1186/s12864-015-2141-9)
Supplement: Additional file 19: Figure S11. — The GO classification analysis of differentially expressed genes (DEGs). (DOC 693 kb) [file 12864_2015_2141_MOESM19_ESM.doc]

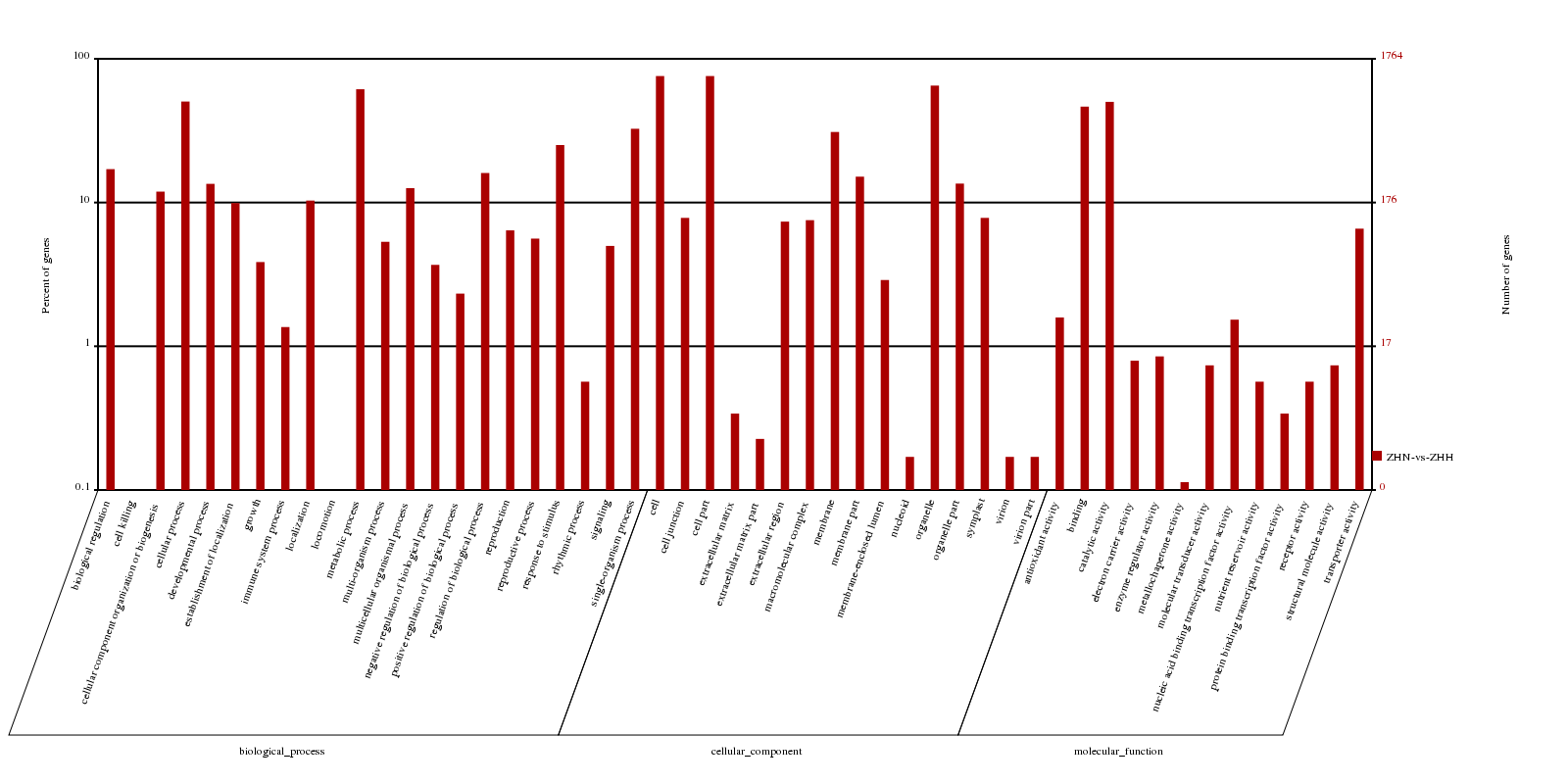


**ZHN-vs-ZHH**


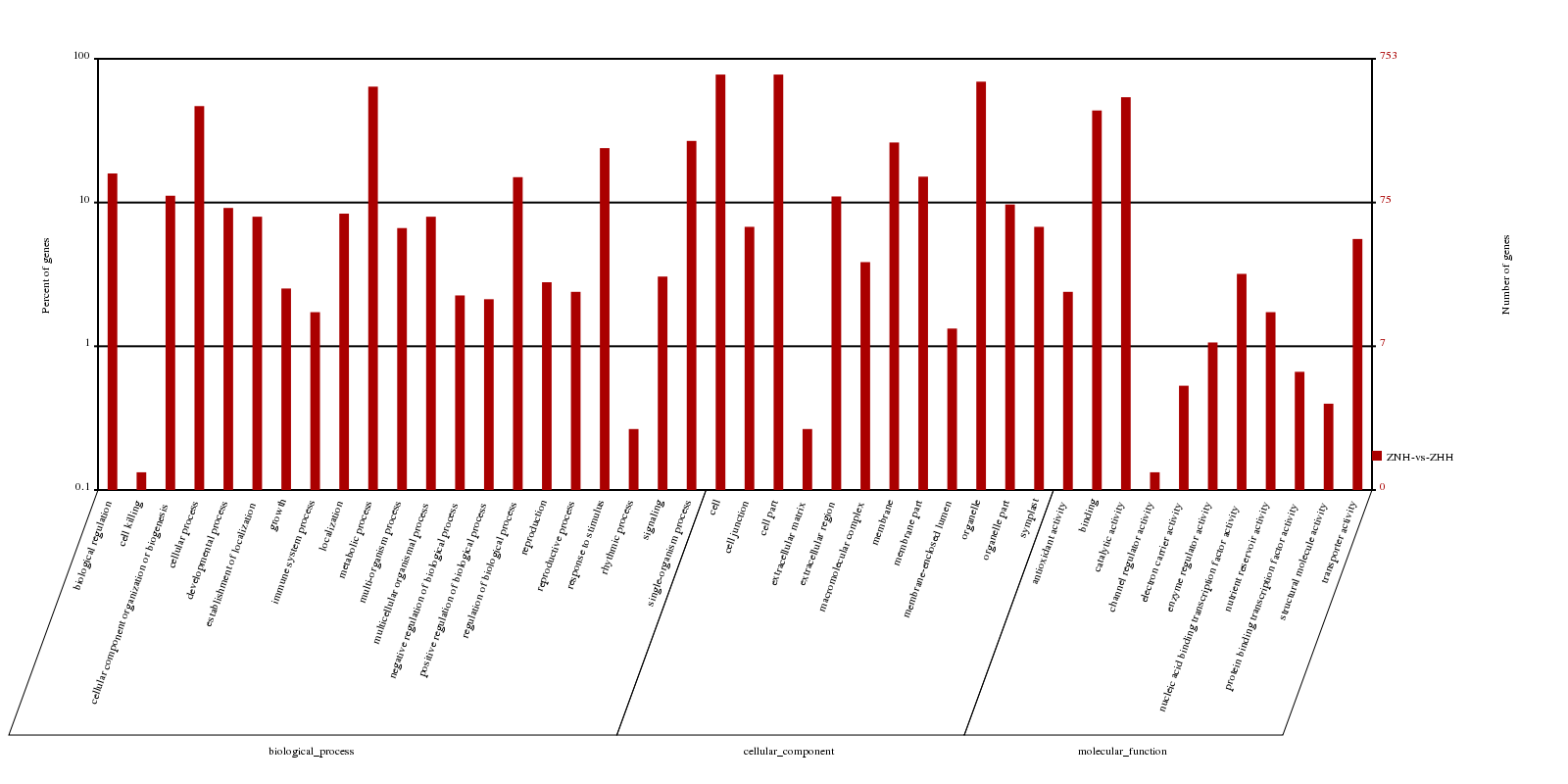


**ZNH-vs-ZHH**


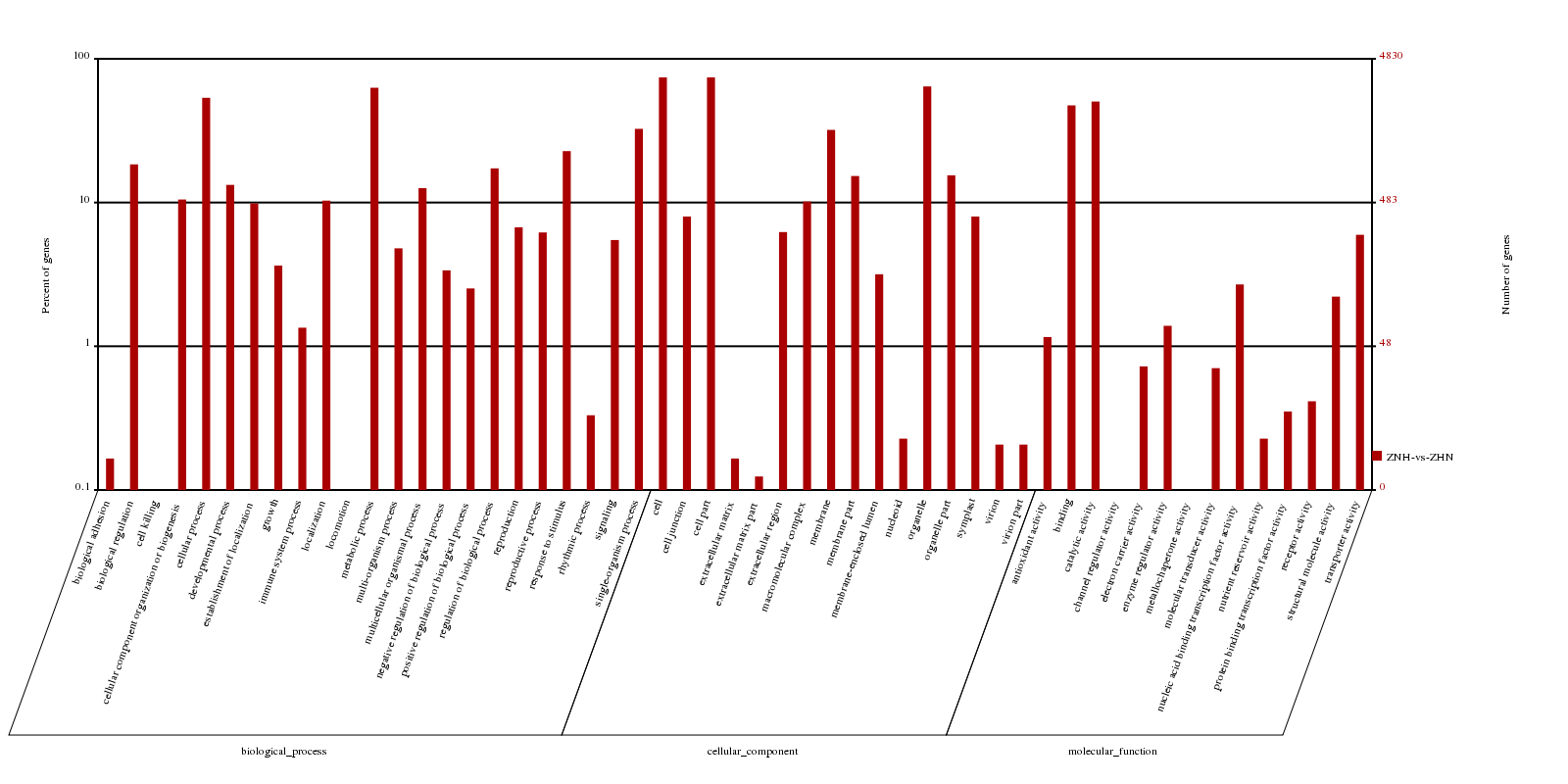


**ZNH-vs-ZHN**


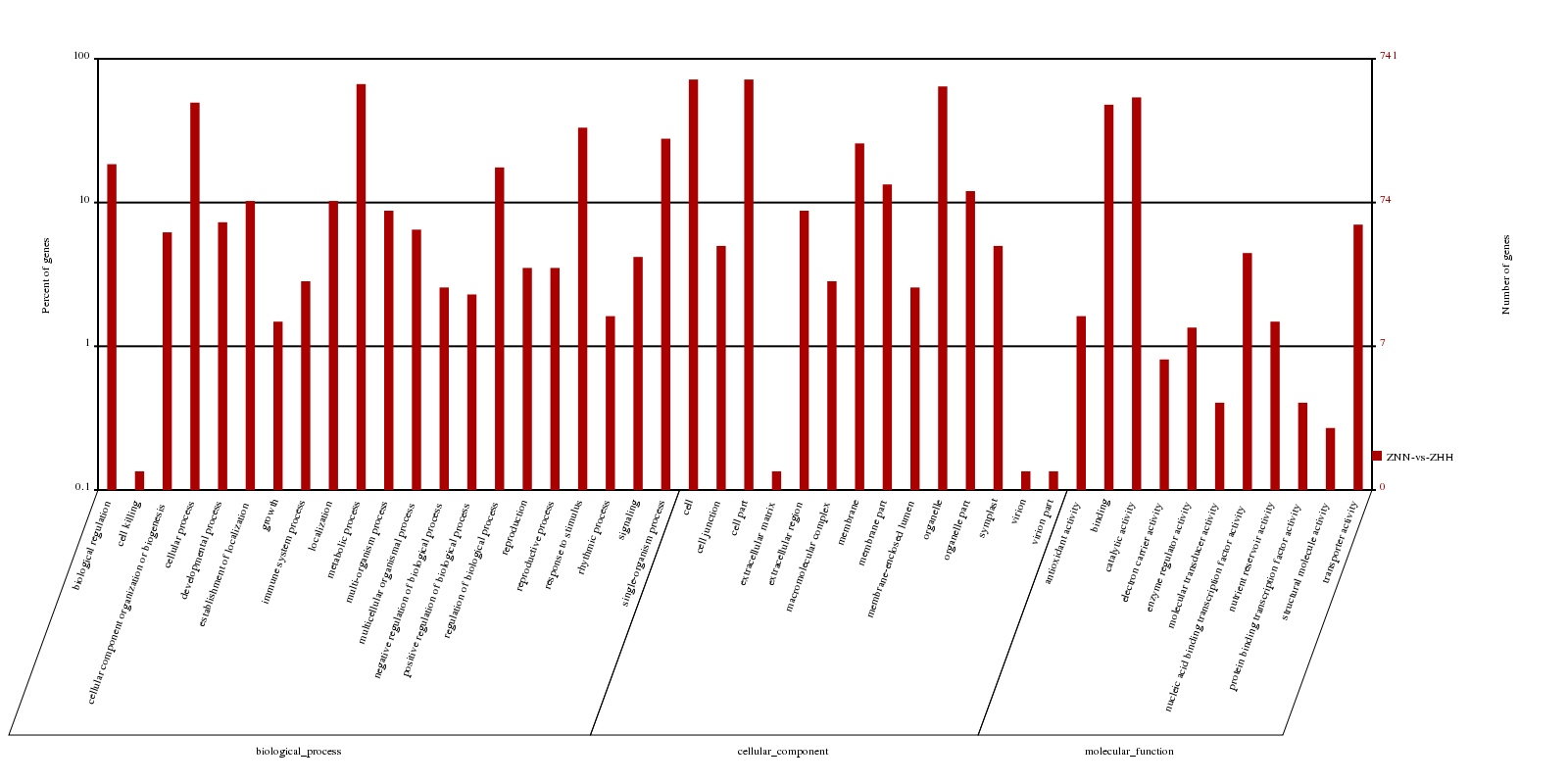


**ZNN-vs-ZHH**


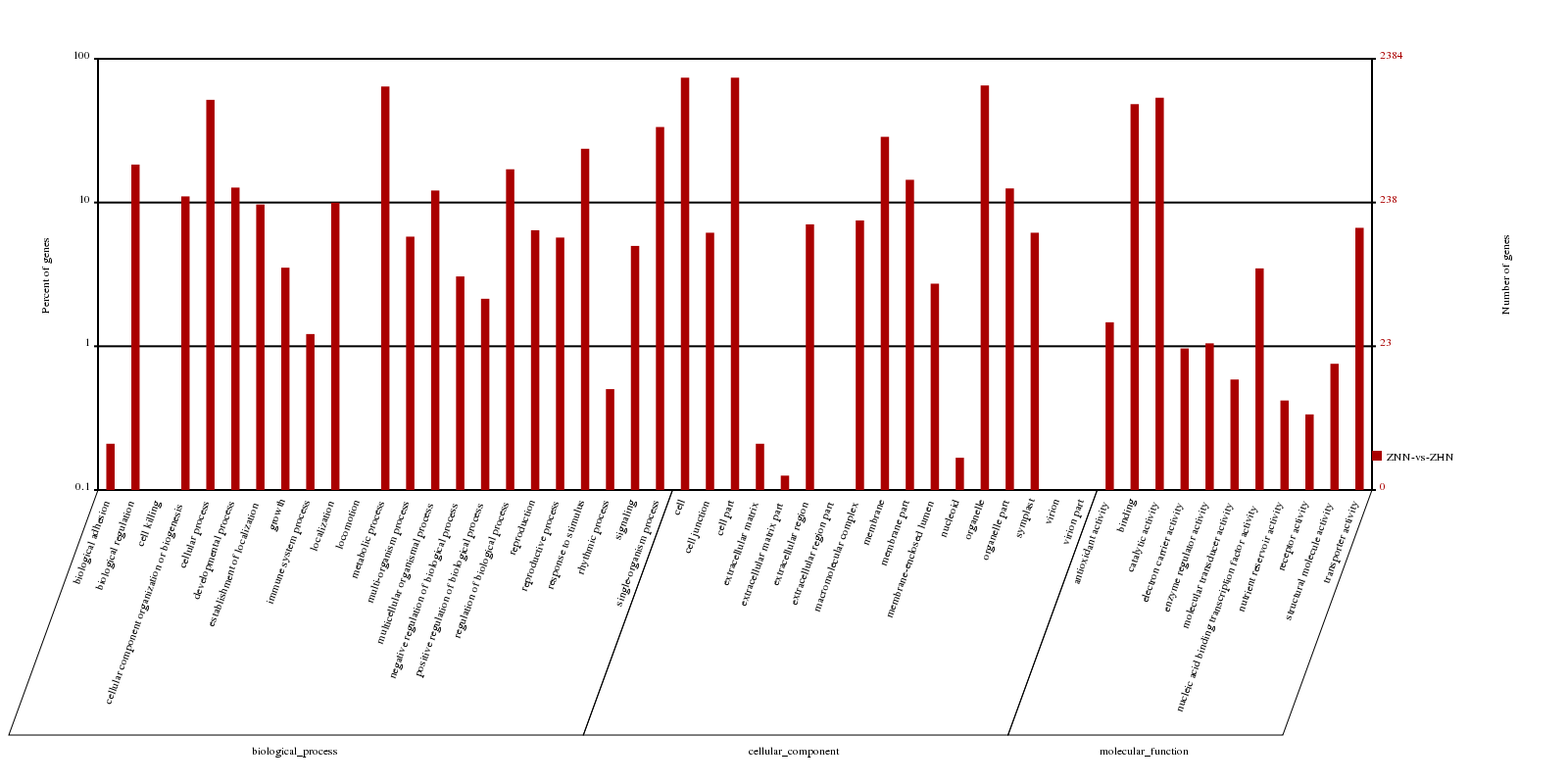


**ZNN-vs-ZHN**


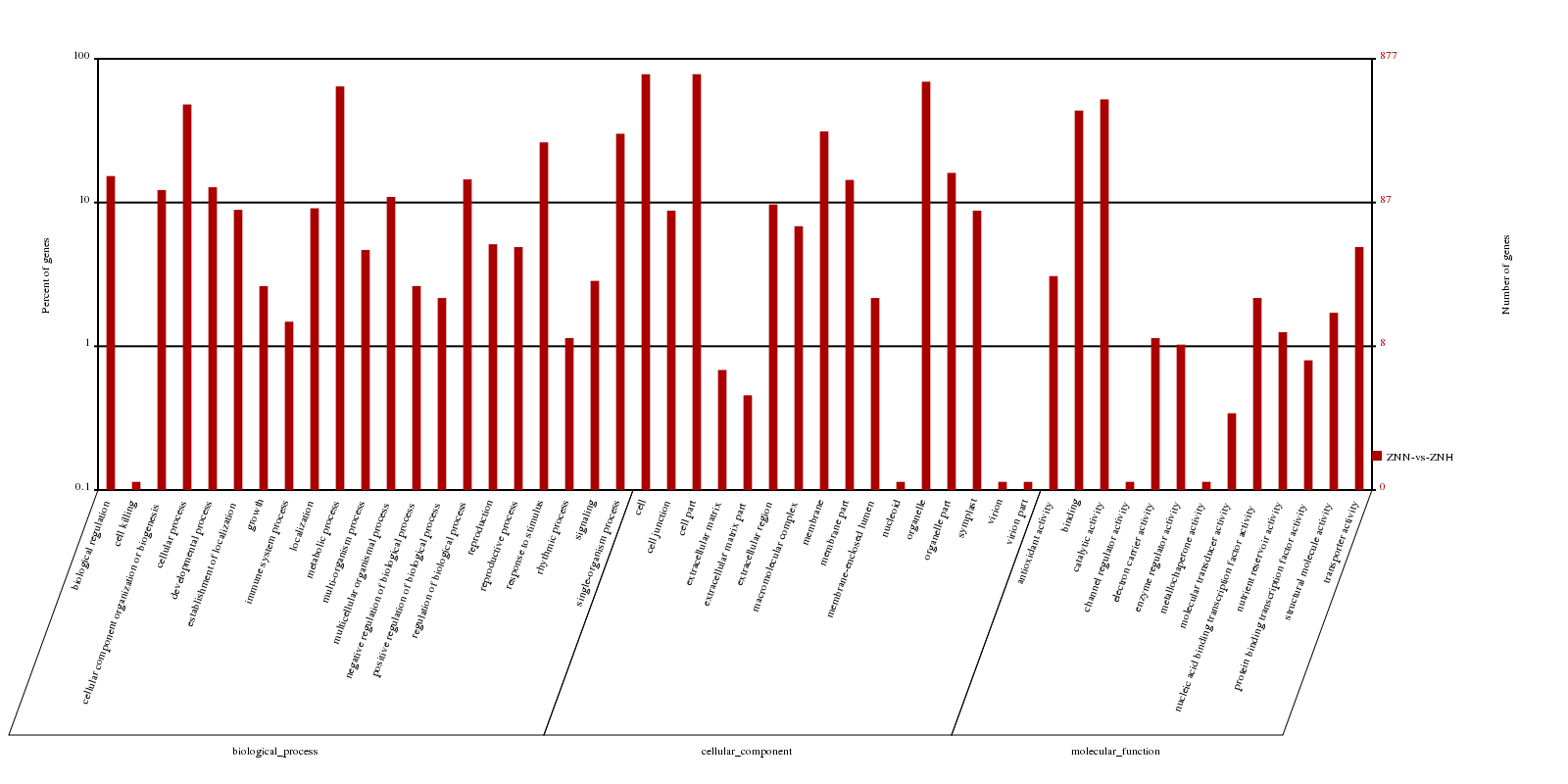


**ZNN-vs-ZNH**

**Fig 11** **GO classification analysis of differentially expressed genes**
